# The Spatial Features and Temporal Changes in the Gut Microbiota of a Healthy Chinese Population

**DOI:** 10.1128/spectrum.01310-22

**Published:** 2022-12-01

**Authors:** Wen Zhang, Na Han, Tingting Zhang, Yujun Qiang, Xianhui Peng, Xiuwen Li, Biao Kan

**Affiliations:** a State Key Laboratory for Infectious Disease Prevention and Control, National Institute for Communicable Disease Control and Prevention, Chinese Center for Disease Control and Prevention, Beijing, China; Huazhong University of Science and Technology

**Keywords:** population studies, gut microbiota, metagenomics

## Abstract

In this study, we aimed to understand the characteristics of the gut microbial composition in a healthy Chinese population and to evaluate if they differed across different regions. In addition, we aimed to understand the changes in the gut microbial composition over time. We collected 239 fecal samples from healthy Chinese adults living in four regions and performed a 1-year time cohort study in a small population in Beijing. The Chinese gut microbiota share 34 core bacterial genera and 39 core bacterial species, which exist in all collected samples. Several disease-related microorganisms (DRMs), virulence factors, and antibiotic resistance genes were found in one or more healthy Chinese samples. Differences in gut microbiota were observed in samples from different regions, locations, individuals, and time points. Compared to other factors, time was associated with a lower degree of change in the gut microbiota. Our findings revealed spatial and temporal changes in the gut microbiota of healthy Chinese individuals. Compared to fecal microbiomes of 152 samples in the publicly released the Human Microbiome Project (HMP) project from the United States, samples in this study have higher variability in the fecal microbiome, with higher richness, Shannon diversity indices, and Pielou evenness indexes, at both the genus and species levels. The microbiota data obtained in this study will provide a detailed basis for further understanding the composition of the gut microbiota in the healthy Chinese population.

**IMPORTANCE** China accounts for approximately 1/5th of the world’s total population. Differences in environment, ethnicity, and living habits could impart unique features to the structure of the gut microbiota of Chinese individuals. In 2016, we started to investigate healthy Chinese people and their gut microbiomes. Phase I results for 16S rRNA amplicons have been released. However, owing to the limitations of 16S rRNA amplicon sequencing, the gut microbiome of a healthy Chinese population could not be examined thoroughly at the species level, and the detailed changes in the gut microbiota over time need to be investigated. To address these knowledge gaps, we started a phase II study and investigated the basis for variations in the gut microbiome composition in a healthy Chinese population at the species level using shotgun metagenomics technology. In the phase II study, we also conducted a time scale analysis of fecal samples from healthy Chinese subjects, as a pioneered study, which quantitatively clarified the changes in the gut microbiota at both the spatial and temporal levels and elucidated the distribution pattern of DRMs in healthy Chinese individuals.

## INTRODUCTION

Population-scale studies of the human microbiome, especially the gut microbiome, such as the Metagenomics of the Human Intestinal Tract (MetaHit) (1), the NIH Human Microbiome Project (HMP) (2, 3), the MicroBiome Quality Control (MBQC) project (4), the National Microbiome Initiative (NMI) (5), and the American Gut Project (AGP) (6), have been conducted in several countries (7, 8). They aid in understanding the relationship between the gut microbiota and health. Gut microbiota disorders are symptomatic or indicative of a predisposing cause of several diseases, such as allergies (9), obesity, diabetes, and even mental illness (10), and they appear to influence cancer immunotherapy treatment (11, 12).

China accounts for approximately 1/5^th^ of the world’s total population. Differences in environment, ethnicity, and living habits could impart unique features to the structure of the gut microbiota of Chinese individuals. Since 2016, the Chinese Pathogen Identification Net, which is a laboratory-based surveillance and early warning network for bacterial infectious diseases, has investigated healthy Chinese people and their gut microbiomes (Chinese Microbiome Project [CMP]) through epidemiological surveys in multiple regions, fecal sampling from healthy people, and examination of the microbiome using sequencing and bioinformatics technology. Phase I CMP results for 16S rRNA amplicon sequencing have been released (13). In the phase I CMP project, we identified several characteristics (age, region, body mass index, physical exercise, smoking habits, alcohol consumption, and yogurt consumption) that influence the gut microbiomes of healthy Chinese people. The distribution patterns of disease-related microorganisms (DRMs) in the gut samples were investigated using 16S rRNA amplicon sequencing; these could be used as markers for the assessment of health risks (13). However, owing to the limitations of 16S rRNA amplicon sequencing, the gut microbiomes of a healthy Chinese population could not be examined thoroughly at the species level. In addition, spatial factors and lifestyle have been proven to influence the human gut microbiome (14, 15); however, the possible effects of these factors over a period of time require thorough investigation. A quantitative and in-depth understanding of the changes in the gut microbiome, especially in the healthy Chinese population over time, and its relationship with other factors is lacking. The detailed changes in the gut microbiota over time and the responsible bacterial species need to be investigated.

To address these knowledge gaps, we started phase II of CMP and investigated the basis for variations in the gut microbiome composition in a healthy Chinese population at the species level, using shotgun metagenomics technology. We conducted a time scale analysis of fecal samples from healthy Chinese subjects. Our findings clarified the changes quantitatively in the gut microbiota at both the spatial and temporal levels. They elucidated the distribution pattern of disease-related microorganisms (DRMs) in healthy Chinese individuals.

## RESULTS

### Common characteristics of fecal samples from healthy Chinese individuals.

**FIG 1:**
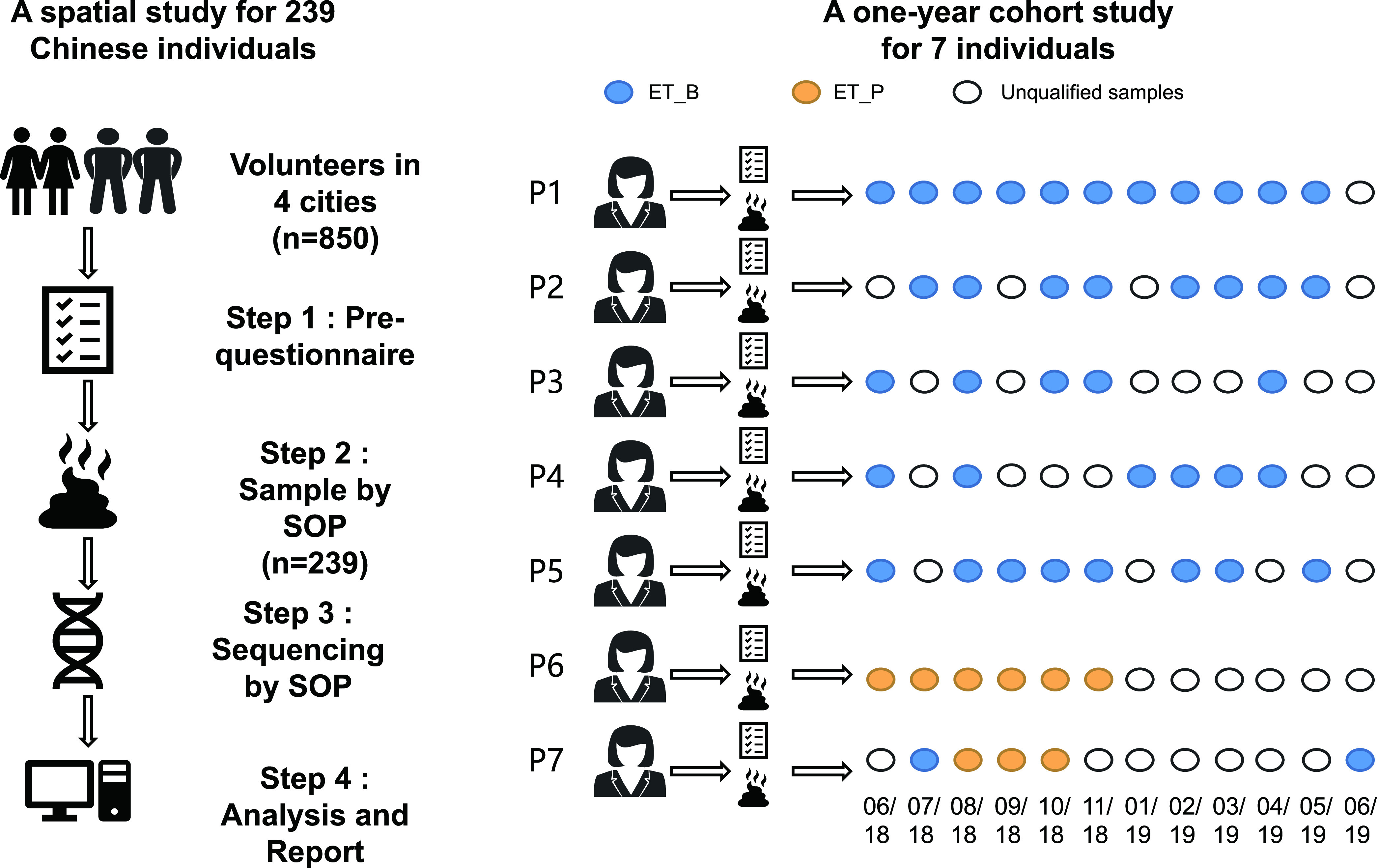
Workflow of the Chinese Microbiome Project (CMP). P, participant; ET, enterotype; B, *Bacteroidetes*; P, *Proteobacteria*.

From 2016 to 2019, we pretested and examined 850 human fecal samples from individuals living in four regions in China (Fig. 1). Detailed information regarding the inclusion/exclusion criteria is provided in Materials and Methods, Table 1, File S1 in the supplemental material. Several factors that could influence the composition of the human gut microbiota were surveyed, including the dietary habits and medical history of the family. The samples obtained from people with high blood glucose levels or high blood pressure were excluded; in addition, we excluded samples from volunteers with a history of drug use, infusion, constipation, or diarrhea in the month prior to sampling. Fecal samples were collected from 239 healthy Chinese volunteers (CMP data set).

**TABLE 1:** Detailed information of samples in the Human Microbiome Project (HMP) and data obtained in this study^*a*^

Group	Samples from HMP	Samples from CMP
No. of sample	152	239
Location	USA	4 regions in China
Age	18 to ~40	18–69
Male:female	NA	1:1.37
Body mass index	18 to ~35	16 to ~30
Blood pressure	<160/100	<140/90
Blood sugar after diet	NA	<11.1 mmol/L
Drug history	No antibiotics in the past 6 mo	No drug in the past mo
Disease history	No pulmonary, cardiovascular, gastrointestinal, hepatic, or renal functional abnormality; no cancer	Absence of 43 kinds of disease (see supplemental material) in the volunteer and in the immediate family
Surgery history	No major surgery in the past 5 yrs	No history of surgery
Disorders	No chronic constipation	No constipation
	No IBD (mild-moderate-severe); No persistent, infectious gastroenteritis, colitis or gastritis, persistent or chronic diarrhea of unknown etiology	No diarrhea in the past mo
	Females who were pregnant or lactating was excluded	Females who were pregnant, lactating or in their menstrual period were excluded
Diet and lifestyle habit survey	NA	Yes
Sequence platform	Illumina PE100	Illumina PE150

a NA, no information supported; IBD, inflammatory bowel disease.

The volunteers were selected from four geographical regions (Beijing [151 samples], Wuxi, Jiangsu province [19 samples], Zigong, Sichuan province [16 samples], and Kaifeng, Henan province [53 samples]). The average age of the volunteers was 28.6 years (range, 18 to 69 years). The male:female ratio was 1:1.37. The mean body mass index (BMI) of the participants was 21.75. Detailed information about the CMP data set is listed in Table S1.

**(i) Taxonomy.** Using the bioinformatics pipeline described in Materials and Methods, we identified 584 bacterial and viral genera in the CMP data set and found some common characteristics in this healthy Chinese population. First, *Bacteroides* organisms were present in all samples (100%), with a corresponding high stability (average percentage, 60%; standard deviation, 23.8). The high percentage of *Bacteroides* in the Chinese gut samples was supported by the reports of previous studies using 16S rRNA amplicon sequencing. Second, 34 bacterial genera were identified as the core genera because they are present in all samples (Table 2). Compared to the 11 core bacterial genera identified using 16S rRNA amplicon sequencing (13), more core genera were identified using the shotgun metagenomic sequencing method. The difference between the two methods could be attributed to the higher resolution of the shotgun metagenomics method for the bacteria in fecal samples and to the use of different databases. Among these 34 core genera, we identified 39 bacterial species that were present in all samples (Table 2). Third, the Shannon-Wiener and Pielou evenness indices, which represent the community diversity in the gut microbiome, were 1.32 to 1.44 and 0.53 to 0.57 (95% confidence interval), respectively, and all of them fit the normal distribution.

**TABLE 2:** List of 34 core bacterial genera and 39 core species in this study

Core genus	Avg percentage (%)	95% confidence interval (%)	Core species
*Alistipes*	4.48	3.85~5.11	Alistipes finegoldii
			Alistipes shahii
*Anaerostipes*	0.62	0.49~0.74	Anaerostipes hadrus
*Bacillus*	0.04	0.039~0.049	
*Bacteroides*	46.53	43.66~49.39	Bacteroides caccae
			Bacteroides caecimuris
			Bacteroides cellulosilyticus
			Bacteroides dorei
			Bacteroides fragilis
			Bacteroides helcogenes
			Bacteroides heparinolyticus
			Bacteroides ovatus
			Bacteroides salanitronis
			Bacteroides thetaiotaomicron
			Bacteroides vulgatus
*Barnesiella*	0.27	0.24~0.30	Barnesiella viscericola
*Bifidobacterium*	1.26	0.77~1.75	
*Blautia*	0.29	0.25~0.33	Blautia hansenii
			*Blautia* sp. strain N6H1-15
*Butyrivibrio*	0.06	0.056~0.069	
Campylobacter	0.13	0.07~0.18	Campylobacter jejuni
*Clostridioides*	0.58	0.51~0.64	*Clostridiales bacterium CCNA10*
*Clostridium*	0.58	0.41~0.75	Clostridioides difficile
			Clostridium bolteae
			Clostridium sporogenes
*Desulfovibrio*	0.45	0.29~0.61	
Enterobacter	0.81	0.24~1.38	
*Enterococcus*	0.06	0.051~0.078	
Escherichia	2.06	1.20~2.92	
*Eubacterium*	2.49	1.55~3.43	Eubacterium hallii
			Eubacterium rectale
			Eubacterium eligens
*Faecalibacterium*	11.70	10.3~13.1	Faecalibacterium prausnitzii
*Faecalitalea*	0.11	0.09~0.12	Faecalitalea cylindroides
*Flavonifractor*	0.37	0.29~0.45	
*Intestinimonas*	0.32	0.26~0.37	Intestinimonas butyriciproducens
*Lachnoclostridium*	0.50	0.39~0.60	Lachnospiraceae bacterium Choco86
			*Lachnospiraceae bacterium* GAM79
			Lachnoclostridium phocaeense
*Lactobacillus*	0.11	0.07~0.16	
*Mordavella*	0.13	0.10~0.15	*Mordavella* sp. strain Marseille-P3756
*Muribaculum*	0.09	0.08~0.10	
*Odoribacter*	2.20	1.90~2.50	Odoribacter splanchnicus
*Oscillibacter*	0.94	0.80~1.08	*Oscillibacter* sp. strain PEA192
*Paenibacillus*	0.06	0.054~0.064	
*Parabacteroides*	2.77	2.46~3.07	Parabacteroides distasonis
			*Parabacteroides* sp. strain CT06
*Prevotella*	6.47	5.18~7.76	
Pseudomonas	0.10	0.077~0.121	
*Roseburia*	1.35	1.11~1.60	Ruminococcus bicirculans
*Ruminococcus*	1.90	1.33~2.47	
Streptococcus	0.63	0.42~0.85	Streptococcus mitis
*Veillonella*	0.13	0.069~0.191	

For the DNA virome in gut microbiota, we did not find any core genus of DNA viruses present in all samples (Fig. S1).

**(ii) DRMs and virulence factors.** In this study, we investigated the distribution of 155 disease-related microorganisms (DRMs) in healthy individuals at the species level based on the “List of Human Pathogenic Microorganisms” (Enacted by China’s Department of Health in 2006) (Table S2). As conditionally pathogenic bacteria, 45 DRMs (29%) were found in one or more healthy samples (Table S2). For example, Enterobacter hormaechei was positive in 16.2% samples from China, Klebsiella pneumoniae, in 46.0%, and Salmonella enterica, in 22.7%. A total of 110 DRMs (71%, Table S2) were not found in any of the samples; this includes Brucella ovis and Chlamydia trachomatis, which are clearly related to infectious diseases and are human health risk markers. This is in line with the results obtained using 16S rRNA amplicon sequencing (13). Using the shotgun metagenomics method, we could clearly evaluate the distribution of DRMs in the gut microbiota on the species level. For example, Vibrio fluvialis could not be distinguished from other Vibrio species, such as V. vulnificus, using the 16S V3-V4 region, but it can be detected using metagenomic methods in this study. Neither *V. fluvialis* nor V. vulnificus were found in the healthy gut samples and belong to group 1 high-risk markers.

Bacterial virulence factors refer to properties such as gene products that enable a microorganism to establish itself on or within a host of a particular species and enhance its potential to cause disease (16). Virulence factors include bacterial toxins, cell surface proteins that mediate bacterial attachment, cell surface carbohydrates and proteins that protect the bacterium, and hydrolytic enzymes that may contribute to the pathogenicity of the bacterium. Based on gene sequences of virulence factors in the VFDB database, we investigated the distribution of 3,583 virulence factors in the gut samples of the CMP data set and found that 9.7% of the virulence factors (348 of 3,583) were present in the gut microbiota of healthy people, covering *Aeromonas*, Campylobacter, Escherichia, Haemophilus, Klebsiella, Salmonella, *Shigella*, Streptococcus, and *Yersinia*.

**FIG 2:**
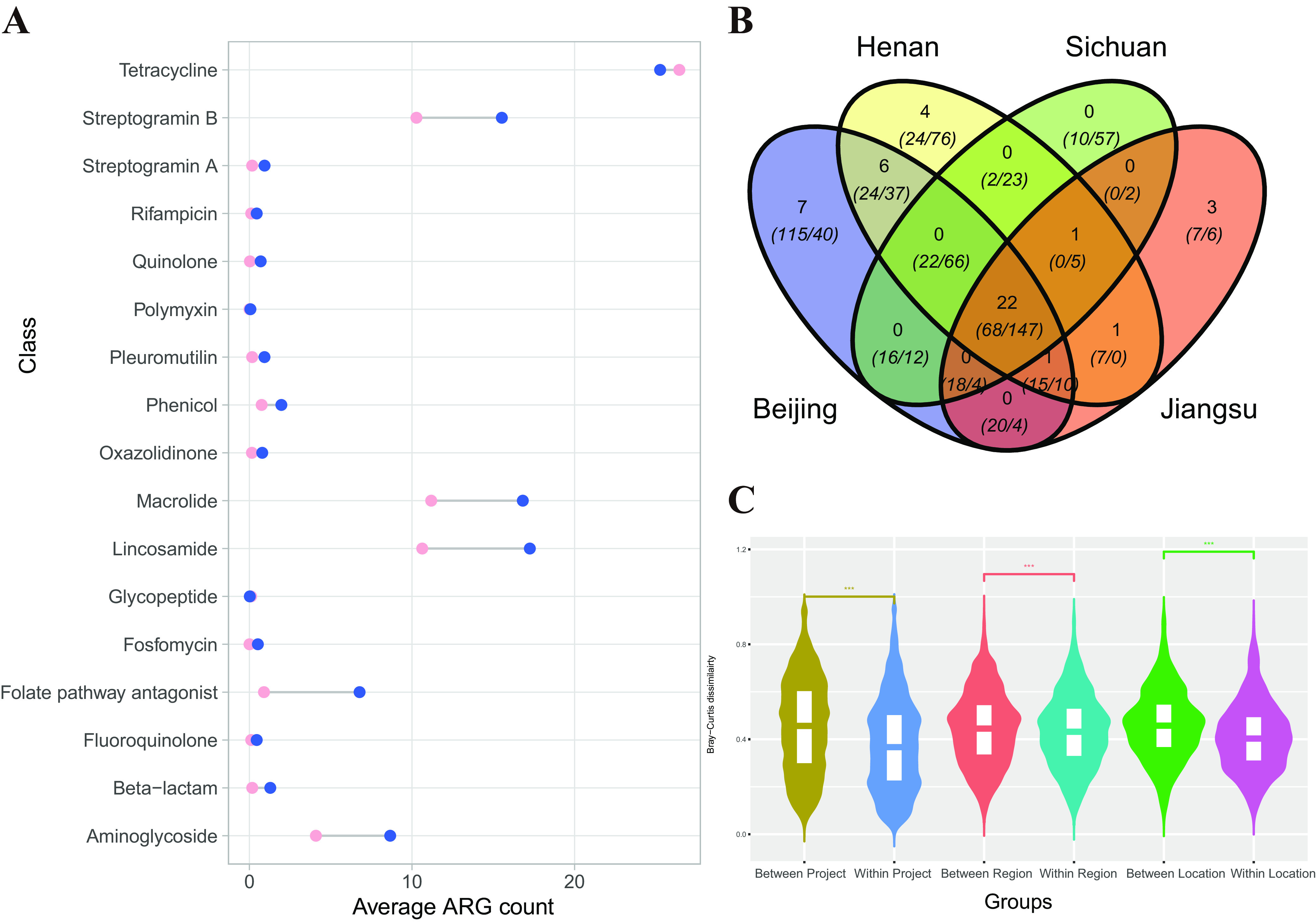
(A) Dumbbell diagram for average ARG number in samples of HMP (pink dots) and CMP (blue dots). (B) Venn diagram for 4 regions. Numbers outside the parentheses represent the identified DRMs. Italicized numbers in parentheses represent the virulence factors and ARGs identified in this region. (C) Violin plots presenting the Bray-Curtis dissimilarity between samples from different groups. “Between project” denotes comparisons between samples from HMP and CMP; “within project” denotes comparisons between samples from the same project; “between region” denotes comparisons between samples from different regions; “within region” denotes comparisons between samples from the same region; “between location” denotes comparisons between samples from different locations; “within location” denotes comparisons between samples from the same location.

**(iii) ARGs.** We also investigated the resistome (total collection of resistance genes against antibiotics) in the gut samples of the CMP data set at the gene level. Among 3,109 antibiotic resistance genes (ARGs) in the ResFinder database (https://cge.food.dtu.dk/services/ResFinder/), 2620 ARGs (84.3%) were not found in any of the healthy samples; these included NDM-1 and its 23 subtypes, which could be used as human health risk markers. A total of 489 ARGs were found in the CMP data set. For example, the aminoglycoside resistance gene (aph2-If_2_AY701528) is common in samples from China (prevalence, 50.4%); this supports the hypothesis that several ARGs have reached normal community transmission in healthy populations and are stable in the gut microbiome. The distinct profiles and our interest in functions related to resistance resulted in the exploration of the resistome in greater detail at the drug class level. We manually curated these 489 genes into 17 drug groups. The tetracycline class was the most abundant ARG in the human gut microbiome (Fig. 2A).

Among the 489 ARGs identified in the CMP data set, 149 ARGs could be assigned to the taxa on the phylum level based on draft genome sequences (metagenome binning) (Fig. S2). Most ARGS (136, 91.3%) were traced to three phyla (*Bacteroidetes*, *Firmicutes*, and *Proteobacteria*). Several ARGs could be identified in bacteria of multiple phyla; for example, the aminoglycoside resistance gene (aph2-If_2_AY701528) could be found in bacteria of *Bacteroidetes*, *Firmicutes*, *Fusobacteria*, and *Proteobacteria*. However, ARGs related to fosfomycin resistance (nine genes) and quinolone resistance (five genes) were only identified in contigs from the *Proteobacteria* phylum.

**(iv) Functional profiles.** Besides identifying virulence factors and ARGs, we also predicted metabolic pathways and their abundance using HUMAnN3 (17). In the CMP data set, we identified 4,095 gene families and 47 KEGG pathways. These pathways mainly cover three categories (metabolism, cellular processes, and genetic information processing) and are closely related to bacterial life activities (Fig. S3).

### Variability of the fecal microbiome with the spatial feature.

In this study, samples from the different regions had different richnesses, Shannon diversity indices, and Pielou evenness indices (Fig. S4A) and clustered by region based on principal-coordinate analysis (PCoA) of the gut microbiota community (Fig. S4E). We found that the DRM patterns of the population from the four regions were also different (Fig. 2B). For 45 DRMs identified in this project (Table S2), only 22 (48.9%) could be found in all four regions, 9 DRMs (20%) were shared by multiple regions, and 14 DRMs (31.1%) were uniquely identified in one region. In addition, only 19.5% of virulence factors and 30% of ARGs were shared by all four regions (Fig. 2B and Fig. S5). We found several ARGs that only exist in a certain region (Fig. 2B and Fig. S5), but with a prevalence of less than 20%.

To evaluate the change of the fecal microbiome on the spatial level, we compared all CMP data set samples one by one and found that the Bray-Curtis dissimilarity between samples from 4 different regions (average, [avg], 0.445; standard deviation [SD], 0.15) was higher than that among the samples from the same region (avg, 0.434; SD, 0.15) (Fig. 2C and Fig. S4C). The significantly higher dissimilarity (Wilcoxon test, *P* < 0.001) supported the effect of spatial features on the gut microbiome on the region level. Samples from the Beijing region could be further divided into group samples from three different locations, labeled BJ1 (53 samples), BJ2 (40 samples), and BJ3 (9 samples). The Bray-Curtis dissimilarities between samples from different locations were also higher than those between samples from the same location, which supports the effect of spatial features—even at the location level—on the microbiota composition (Fig. 2C and Fig. S4D).

The effect of spatial features on the gut microbiome could also be supported by comparison with public data. Compared to fecal the microbiomes of 152 samples publicly released in the HMP project from United States (2, 3), samples in this CMP project have higher variability in the fecal microbiome, with higher richness, Shannon diversity indices, and Pielou evenness index, at both the genus and species levels. By calculating the Bray-Curtis (BC) dissimilarity between pairs of samples, we found that samples of the Chinese volunteers had higher average BC dissimilarity than those from the HMP (Fig. S4B; Wilcoxon test, *P* < 0.001), which supports the higher level of variability in the fecal microbiome in the Chinese population.

To further evaluate the shared proportion of the CMP data set on the sequence level, we generated a microbiome genetic similarity (GS) value to represent the overlap between samples, which is described Materials and Methods. We found that the average GS for human gut microbiota is 0.068. We also found that samples from the same region or the same location have a higher GS value than that from different cities (Fig. S4F), which represents the sequence variability of the fecal microbiome caused by the spatial feature.

### Temporal changes in the fecal microbiome.

To compare the effects of the temporal features on the human gut microbiome, we conducted a 1-year cohort study in a small population from Beijing (Fig. 1); we collected fecal samples from seven individuals from 2018 to 2019 at 12 time points, with each time point corresponding to a month (Fig. 1). At each sampling time, the seven volunteers were assessed; height, weight, blood pressure, and blood glucose levels were measured on-site every month, and a detailed report on candidate factors affecting the gut microbiome was constructed. Finally, 49 fecal samples were collected and sequenced (Fig. 1). The sample and quality control process is described in Materials and Methods.

**FIG 3:**
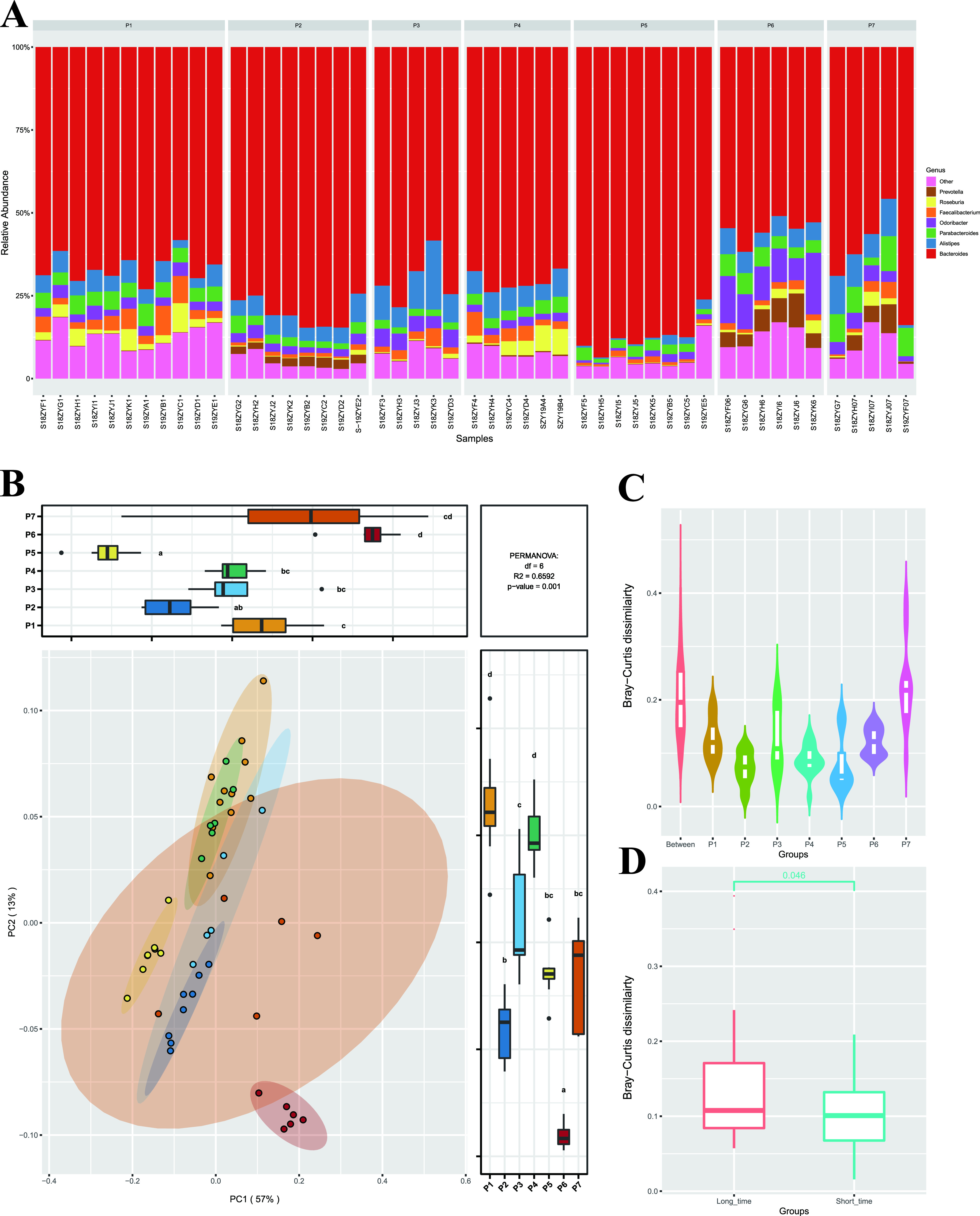
(A) Percentage of different bacterial genera in fecal samples from seven participants. (B) PCoA diagram of samples from seven participants. In the boxplots, the letters a, b, and c to show statistically significant differences between groups. For all groups with the same letter, the difference between the means is not statistically significant. If two variables have different letters, they are significantly different. (C) Violin plots presenting the Bray-Curtis dissimilarity between samples from different groups. “Between” group denotes comparisons between samples from different participants; the P1 to P7 group denotes comparisons between samples from the same participant. (D) Boxplots presenting the Bray-Curtis dissimilarity between samples from different groups. “Short-time” represents a short time interval, where the samples were collected in two consecutive months. The “long-time” interval is an interval longer than 6 months.

After analyzing 49 fecal samples collected from the volunteers at 12 time points and identifying the microbiota to the genus and species levels, we observed that the percentages of bacteria in different samples from the same volunteer were not stable over time (Fig. 3A). Of 227 genera identified in these 49 samples, 155 (68.3%) were positive in only one or very few time point samples, not in all time points, which supported the healthy gut microbiota containing a certain proportion of transient bacterial strains. These transient bacterial strains could come from the outside, such as food or the human-external environment, which needs to be further explored in a later study. Even for those core genera existing in all collected samples, a fluctuation of relative amount still existed. We used the biological coefficient of variation value to estimate the range of dispersion of the relative abundances of the bacteria. Basically, a higher coefficient of variation value represents greater volatility in positive samples. *Prevotella*, which was detected in all the samples, was the most variable core genus with high coefficient of variation value (0.61). The percentage of *Prevotella* fluctuated over time (Fig. 3A), which would cause the change of enterotype (Fig. 1). Samples from participant 1 (P1), P2, P3, P4, and P5 were all assigned to the ET_B enterotype, while samples from P6 were assigned to the ET_P enterotype. P7 had two enterotypes (ET_B and ET_P) (Fig. 1 and Fig. 3A).

The gut microbiome is not always stable over time; however, the level of change is low. The BC dissimilarity between samples originating from the same individual were significantly lower than that between samples originating from different individuals (Fig. 3C; Wilcoxon test, *P* < 0.001). PCoA supported this finding; samples from the same individuals were clustered. Permutational multivariate analysis of variance (PERMANOVA) indicated that the effect caused by the individual (*R*^2^ = 0.6592; Fig. 3B) was higher than that caused by spatial factors (*R*^2^ = 0.0339).

We calculated the Bray-Curtis dissimilarity between the samples collected at different time points for each individual. Based on the interval between two sampling time points, we divided the comparisons between samples from the same individual into two groups (short and long term). Short-term represents a short time interval in which the samples were collected in two consecutive months. A long-term interval is an interval longer than 6 months. Based on the calculated Bray-Curtis dissimilarity for samples and comparison between groups, it was found that the differences in the gut microbiota between samples increased gradually with time. The Bray-Curtis dissimilarity in the short-term interval group was significantly lower than that in the long-term interval group (Wilcoxon test, *P* < 0.05) (Fig. 3D).

In this 1-year cohort study, we also used sourmash (18) to estimate the shared proportion of bacteria remaining in the samples across two time points on the sequence level. We observed that the GS value of two samples from the same owner (avg, 0.245) was significantly higher than that of samples from different people (avg, 0.08; Fig. S4F; Wilcoxon test, *P* < 0.001). Although this GS value would slightly decrease with time, for a long-terms sample (>6 months), the average GS value was still 0.226, significantly higher than that between people (Fig. S4F; Wilcoxon test, *P* < 0.01), which also supported the finding that samples from the same person always had a higher similarity than samples from different people.

In this 1-year cohort study, we also identified 21 DRMs among 49 fecal samples on the species level. Of these 21, 12 DRMs (57.1%) were stable in one or more people. We did not find any stable existing bacterial virulence factors that were present in >60% of samples from the same individual. This finding supports the hypothesis that the existence of virulence factors is transient rather than stable in the gut microbiome.

We also investigated the resistome stability on a time scale using these 49 samples from 7 individuals. Among the 49 samples, 182 ARGs were identified; 13 ARGs (7.1%) were stable in all 49 samples from the 7 individuals; 60 ARGs (33.0%) were identified in all samples from a single person. These 73 ARGs were called time-stable ARGs. The other ARGs (59.9%) were only transiently positive in one or more time point samples; this supports the partially stable nature of the ARGs in the human gut microbiome.

The time-stable ARGs exhibited differences in their occurrence in the samples from different people. The beta-lactam ARG (blaOXA-85_1_JANA01000064) was positive in all five samples from P7 but was not found in any other samples. The aminoglycoside ARG [aph(3″)-Ib_5_AF321551] is a time-stable ARG in P4 and P5 but was transient in P6.

For 182 ARGs identified in 49 samples from 7 individuals, 105 ARGs could be assigned to the taxa on the phylum level. We found that the tetracycline ARG (tet32_2_EF626943) is a time-stable ARG in P5, but it was from a bacterial strain assigned to the *Bacteroidetes* at the phylum level at the *T*4 (September 2018) sample point but to the *Firmicutes* at the *T*11 point (May 2019), which supported the idea that some ARGs may also come from different bacterial strains.

### Variability of the fecal microbiome with lifestyle.

**FIG 4:**
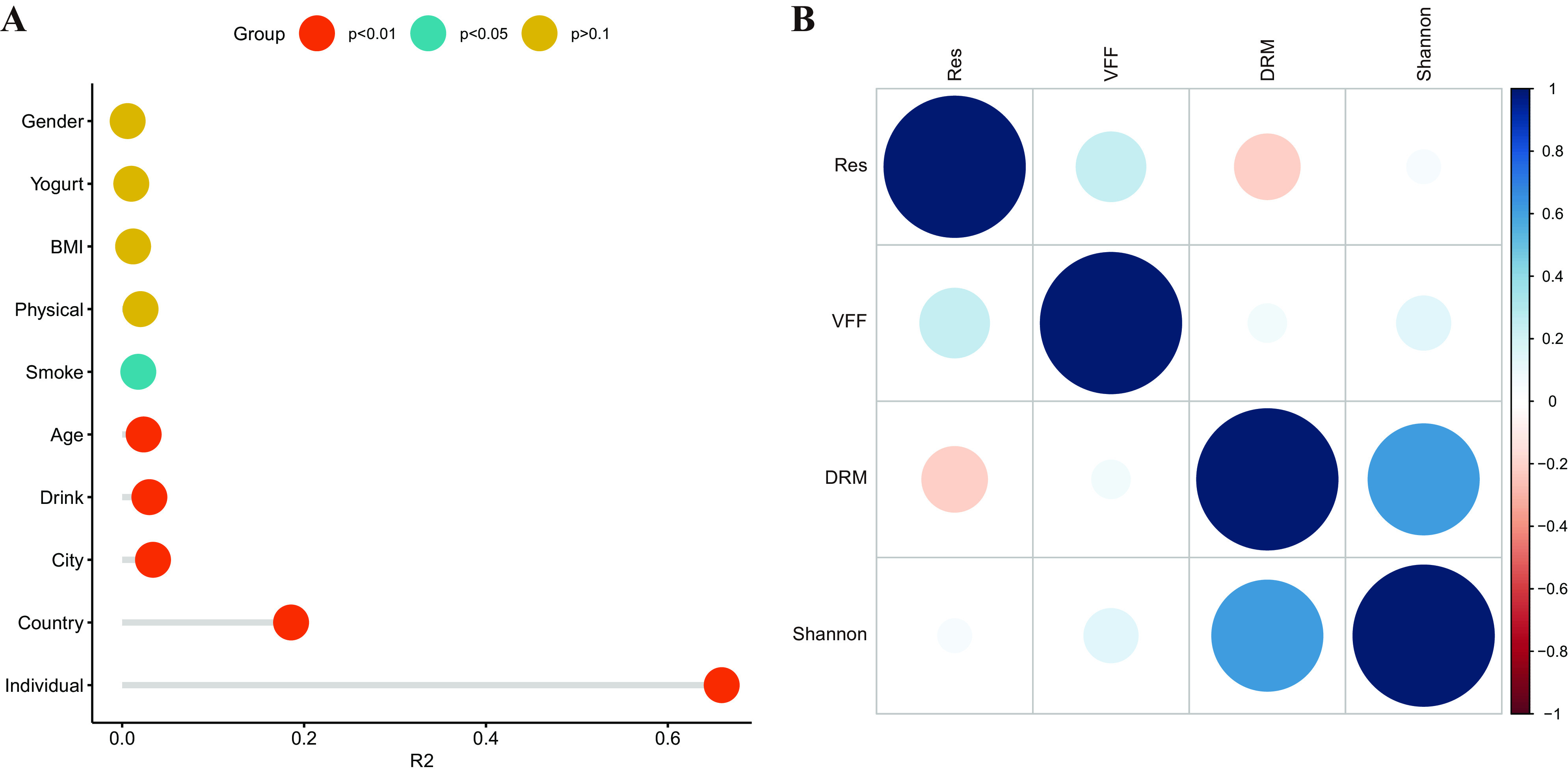
(A) Lollipop chart for PERMANOVA results for the different associations between the variations in gut microbiota and host characteristics. (B) Bubble diagram for the correlation between DRMs, ARGs, virulence factors, and Shannon index.

We used PERMANOVA to explore the associations between gut microbiota and host characteristics. We found that individuals exerted the strongest effect (Fig. 3B and 4A), exceeding the effect of other host factors. Among the different spatial factors, the country/project level has the strongest effect; this was supported by the results of He et al. (19).

In this study, volunteers who consumed yogurt every day or often (more than 3 times in the past month) were grouped as “yogurt+,” while those who never ate yogurt were classified as “yogurt–.” Yogurt+ people had a higher percentage of *Bifidobacterium* spp. than those in the Yogurt– group (Fig. S6).

In this study, we did not find any significant difference between the male and female participants with respect to the gut microbiome richness; however, the female individuals had a higher percentage of *Bifidobacterium* spp. than the male individuals (Fig. S7).

Alcohol consumption is associated with health risks. In this study, people who consumed alcohol had a significantly higher (Wilcoxon test, *P* < 0.05) Shannon index than those who did not. We used linear discriminant analysis effect size (LEFSE) to distinguish the marker genera between the drink+ and drink– groups. In the drink+ group, the sample had a higher percentage of Enterobacter spp., Klebsiella spp., and *Desulfovibrio* spp.

We also compared the identified DRMs, virulence factor numbers, ARGs, and gut microbiota Shannon index in this study; however, we did not found a correlation between virulence factors and ARGs (Fig. 4B).

## DISCUSSION

### Update in CMP phase II.

In the previously published CMP phase I study, we investigated the gut microbiota characteristics and patterns associated with disease-related microorganisms in a population of healthy Chinese individuals using the 16S metagenomic method. In phase II, we updated the method to include shotgun metagenomics methods and raised the resolution to the species level. Compared to the 11 core genera identified in phase I using the 16S method (13), 34 core genera and 39 core bacterial species were identified using shotgun metagenomics methods (Table 2). The difference between the two methods could be attributed to the higher resolution of the shotgun metagenomics method for the bacteria in fecal samples and to the use of different databases. Owing to the lack of reference genome sequences for *Coprococcus*, *Gemmiger*, and *Parasutterella* in the shotgun metagenomics database, they were not identified using the shotgun metagenomics method. With the exception of these three genera, all other core genera identified using the 16S method were identified as core genera using the shotgun metagenomics method.

Using the shotgun metagenomics method, we could clearly evaluate the distribution of DRMs in the gut microbiota. Vibrio fluvialis could not be distinguished from other *Vibrio* species, such as V. vulnificus, using the 16S V3-V4 region, but it can be detected using metagenomic methods based on genome sequences. Other common DRMs, such as Campylobacter jejuni and Salmonella enterica, were found in several samples from people without any diarrhea symptoms in the month before sampling. Therefore, the existence of these DRMs, such as Campylobacter jejuni and Salmonella enterica, should not be directly used as risk markers but should be considered comprehensively, taking into account the abundance and type of DRM and the presence of virulence factors.

In contrast to the 16S method, shotgun metagenomics enables the understanding of the existence of patterns of DNA viruses and archaea in gut samples from healthy Chinese populations. The DNA virome was different between the CMP and HMP samples (Fig. S1). Sk1 virus and T4 virus are common in most HMP samples, but not in CMP samples. However, a similar divergence between countries was not observed for archaea.

In CMP phases I and II, we determined the effects of spatial factors and lifestyle on the gut microbiota composition. The impact of spatial features on the fecal microbiome at the levels of country, region, and location were supported by the results from this study; samples from different countries/regions/locations exhibit higher divergence than those from the same places. Compared to the HMP data set, samples in this study have higher variability in the fecal microbiome, with higher richness, Shannon diversity indices, and Pielou evenness index, as well as having more ARGs in several classes (Fig. 2A). Besides the spatial factor, the different microbiota and resistomes between CMP and HMP may also be caused by the different sampling times of two projects.

In CMP phase II, we investigated the changes in the gut microbiota in a healthy Chinese population over time. Analyzing the gut samples from the same individuals for 1 year, we found that the gut microbiota community from the same people always has a higher similarity than that from different people, which supports community stability over time. However, in samples from P7, the enterotype changed with time. The Bray-Curtis dissimilarity for the short-term interval groups was significantly lower than that for the long-term interval groups (Fig. 3D; Wilcoxon test, *P* < 0.05).

### Potential application of gut microbiota to evaluate the health status of the participants and trace the host source.

“Healthy” is not equivalent to the complete absence of disease-causing microorganisms (DRMs). In this study, we found that even for people without any disorder symptoms, 45 DRMs, which are conditionally pathogenic bacteria, were detected in healthy volunteers, and 12 DRMs were found to be stable, existing in gut samples from the same people sampled at different time points. Based on phase II findings for healthy Chinese individuals, we released a program (GuthealthyWGS) for users to quickly obtain information about their DRMs and virulence factors based on the metagenomic results of their gut samples (https://github.com/zhangwencdc/GuthealthyWGS.git).

“Healthy” gut microbiota is not equivalent to the “same.” One important objective for this field is to make the health status measurable and comparable. Based on the information available for the volunteers in this study and the Mantel test, we compared the influence of ten factors on gut microbiota (individual, country, region, alcohol consumption, age, smoking, physical activities, BMI, yogurt consumption, and sex). The most influential factor was the individual (the host of the samples), followed by the country (the sample project HMP or CMP) and region (sample location). Physical activities, BMI, yogurt consumption, and sex were not found to have a significant impact on gut microbiome richness (Fig. 4A; *P* > 0.1); however, these factors influenced the occurrence of specific species, such as *Bifidobacterium* spp.

Individuals are the main factor influencing the gut microbiota; the samples from the same individual have higher similarity than those from different individuals. We also found differences in the gut microbiota composition with time; the relatively small change in the human gut microbiota over a short period (<1 month) enhanced its potential for use in host source traceability in the future. It is noteworthy that the conclusion about the host of the samples being the most influential factor is only based on samples from seven individuals, whereas the rest of the factors are based on a big data set. Thus, we still need more work to support this conclusion, as well as more data to make it clearer for the temporal changes in the gut microbiota. Our study also had the limitations of having only four sample regions and employing only DNA sequencing data. In our future studies, we will process a larger number of samples and also evaluate RNA sequencing data. These limitations notwithstanding, our results have helped establish that the characteristics of gut microbiota change with time and space.

**Conclusions.** In summary, our findings revealed changes in the Chinese gut microbiota over time and spatial levels and examined the distribution patterns of disease-related microorganisms (DRMs) at the species level. The microbiota data obtained in this study on the species level will provide a detailed basis for the healthy gut microbiome composition in the Chinese population.

## MATERIALS AND METHODS

### Samples collection in four regions.

From 2016 to 2019, 239 fecal samples were sampled from people living in four cities in China (Beijing, Wuxi, Kaifeng, and Zigong). These four regions are located in the northern, southern, central inland, and western regions of China, respectively. All volunteers in the groups were given a pretest and a detailed questionnaire about their age, career, drug/medical history of both themselves and their immediate families, and their living habits (smoking, physical exercise, and fruit and alcoholic drink consumption) (Table S1) to determine the health level of the volunteers. We also measured the height, weight, blood pressure, and blood glucose levels on-site and constructed detailed inclusion/exclusion criteria to filter out candidate factors affecting the microbiome. Detailed information about the questionnaire process and inclusion/exclusion criteria is provided in the supplemental material.

Briefly, only adult (age 18~69) volunteers were included in this project (Table 1). People with any symptoms of diarrhea, constipation, bloody stool, or cold infection in the month prior to sample collection were excluded. People who took any drugs, oral or injectable, in the past month were also filtered out. By determining the medical history of volunteers and that of their immediate family, people with any of 43 kinds of diseases were also excluded (listed in the supplemental material., personal information questionnaire). For evaluation of blood pressure and blood glucose levels on-site, only volunteers with a blood pressure value below 140/90 mmHg and 11.1 mmol/L blood glucose levels at the time of the test were included for further study.

All fecal samples were collected within 24 h after finishing the pretest and questionnaire. The feces were collected from a disposable bedpan given to the volunteers and were not obtained from a flush toilet, to avoid the complication of collecting toilet water. Samples were stored in ice boxes and transported to the laboratory within 4 h. The study was approved by China CDC; all experiments were performed in accordance with relevant guidelines and regulations, and all people provided informed written consent.

### Sample collection in a 1-year cohort study.

Each month from June 2018 to June 2019, we collected fecal samples from 7 healthy Chinese individuals residing in Beijing, China. The eligibility criteria for participation in this study and the sampling process were same as described in the previous section. Details regarding the characteristics of the subjects are provided in the supplemental material. In total, 49 fecal samples from 7 individuals were collected, and DNA was extracted and successfully sequenced. The number of samples collected from each subject, corresponding to a total of 12 time points, varied in the range 5 to 11 (Fig. 1).

The daily habits (smoking, physical exercise, and fruit and alcoholic beverage consumption) of the subjects were also evaluated each month to determine their health status. Additionally, their heights, weights, blood pressure values, and blood glucose levels were recorded on-site every month. Thereafter, we constructed a detailed report on candidate-specific factors that could affect their gut microbiome. Detailed information in this regard is provided in the supplemental material, personal information questionnaire.

### DNA extraction, sequencing, and quality control analysis.

DNA was extracted from the samples within 24 h using the QIAamp Fast DNA stool minikit (Qiagen, Germany), as per the manufacturer’s instructions.

The shotgun metagenomics library was built using the TruePrep DNA library prep kit for Illumina and sequenced using an Illumina HiSeq platform with paired-end sequencing generating reads of 150 bp. Using FastQC (20) and Fastp (21), we filtered out low-quality reads. The low-quality (<Q20) bases at the end of the reads were trimmed off, and only reads with ≥100 bp were retained as high-quality reads. The number of high-quality reads for each sample was above 10^7^, and the percentage of bases with values higher than a quality value of Q30 was above 80%. After quality control of raw reads, we removed the human sequences using Bowtie 2 (22) (human database version: GRCh38).

The public fecal microbiome data used in this study were downloaded from the HMP database (http://www.hmpdacc.org/). After filtering out samples without metadata, only samples from healthy individuals and those released with shotgun metagenomics data of >100,000 reads were kept for further analysis. Finally, we got raw sequencing reads from 152 healthy individuals in the HMP project.

### Analysis pipeline for samples with Shotgun metagenomics method.

The analysis pipeline used in this study was released in GitHub (https://github.com/zhangwencdc/GuthealthyWGS).

For the qualified samples, the relative abundances of humans, bacteria, viruses, and archaea were calculated using Kraken 2 (23) based on database minikraken2_v2_8GB_201904_UPDATE. The core genera in this study are defined to exist in all samples with an incidence of ≥0.01%.

The variability in the relative abundance of bacteria in the samples was determined by calculating the Bray-Curtis dissimilarity for each individual over time using the R package vegan. The data obtained were presented using the R packages, ggplot2 (24) and ggsignif (25). Significant differences between groups were determined using the Wilcoxon test, and significance was set at *P* < 0.05.

The genetic similarity in this study was calculated after filtering out sequences from the humans, bacteria, viruses, and archaea. For each sample, we took 10,000 31-kmer sequences at random and compared them one by one using sourmash (18). For any two samples, the comparison was repeated 10 times. The genetic similarity between these two samples was calculated using the following formula:

genetic similarity (GS) = (N1 + N2 +…+ N10)/Nsum

*N*1 is the number of same kmer sequences between two samples for the first comparison; *N*2, *N*3, …, *N*10 is the number of same kmer sequences between two samples for the next repetition of calculations; *N*sum is the total number of Kmer sequences for 10 repetitions (*N*sum = 100,000).

The microbiome of human fecal samples was analyzed using HUMAnN3 (v3.1.1) (17) with the UniRef 90 and KEGG databases to characterize composition and function. After normalizing to copies per million reads, the abundance and coverage of each pathway was identified in each community (coverage, >5%) and presented using the R package, ggplot2 (24).

### Detection of DRMs and ARGs.

The DRMs identified in this study represent the bacterial species that cause or might potentially cause disease in humans or in other bacterial species in the same genus as those that have been proven to cause disease. Based on the “List of Human Pathogenic Microorganisms” (enacted by China’s Department of Health in 2006), we listed 155 DRMs with genomes which covered Chinese national legal infectious diseases and several pathogens detected in recent outbreaks as well as several novel bacterial species proven to cause disease; all listed DRMs are listed in Table S2. To detect the existence of DRMs, all shotgun metagenomics sequences were classified using the Kraken 2 method (23) based on the minikraken2_v2_8GB_201904 database, and only DRM species with a percentage of >0.1% in at least 1 sample were retained for further analysis.

Virulence factors were identified using the Bowtie 2 method (22) based on the VFDB database (16). Only alignments with identity greater than 80% were kept for further analysis. The total match region was calculated for each virulence factor, and only coverage (coverage percentage = total match region/gene size) greater than 90% was considered a positive result.

The same pipeline was used for detection of ARGs. The ResFinder database was used (https://cge.food.dtu.dk/services/ResFinder/). The analysis script was released in GitHub (https://github.com/zhangwencdc/GuthealthyWGS).

The correlation between the presence/absence of virulence factors and ARGs was done using the R package corrplot (26).

The metagenome binnings were assembled using SPAdes v3.13.0 (27) and then performed using MetaBAT v2.12.1(28). For taxonomic classification of metagenome binnings, all assembled contigs were classified by utilizing Kraken 2 (23) software based on the minikraken2_v2_8GB_201904 database. We kept this taxonomic result for further analysis only when more than 50% of contigs in the same metagenome binnings had the same taxonomic classification results.

### Ethics approval and consent to participate.

Written informed consent was obtained from participants before enrollment in this study. The study was approved by China CDC, and all experiments were performed in accordance with relevant guidelines and regulations.

### Data availability.

All sequencing data in the project, as well as metadata (Table S1), were deposited in the NCBI SRA database (BioProject no. PRJNA801051, SRA no. SRR17790811 to SRR17791038 and SRR21777554 to SRR21777564).
