# Supplementary material for: The Spatial Features and Temporal Changes in the Gut Microbiota of a Healthy Chinese Population
Source: Microbiol Spectr. 2022 Dec 1;10(6):e01310-22. doi: 10.1128/spectrum.01310-22 (PMC9769860; doi:10.1128/spectrum.01310-22)
Supplement: Supplemental file 1 — Supplemental material. Download spectrum.01310-22-s0001.pdf, PDF file, 3.3 MB [file spectrum.01310-22-s0001.pdf]

Supplemental Figure 1. The heatmap of DNA virome in CMP and HMP projects.

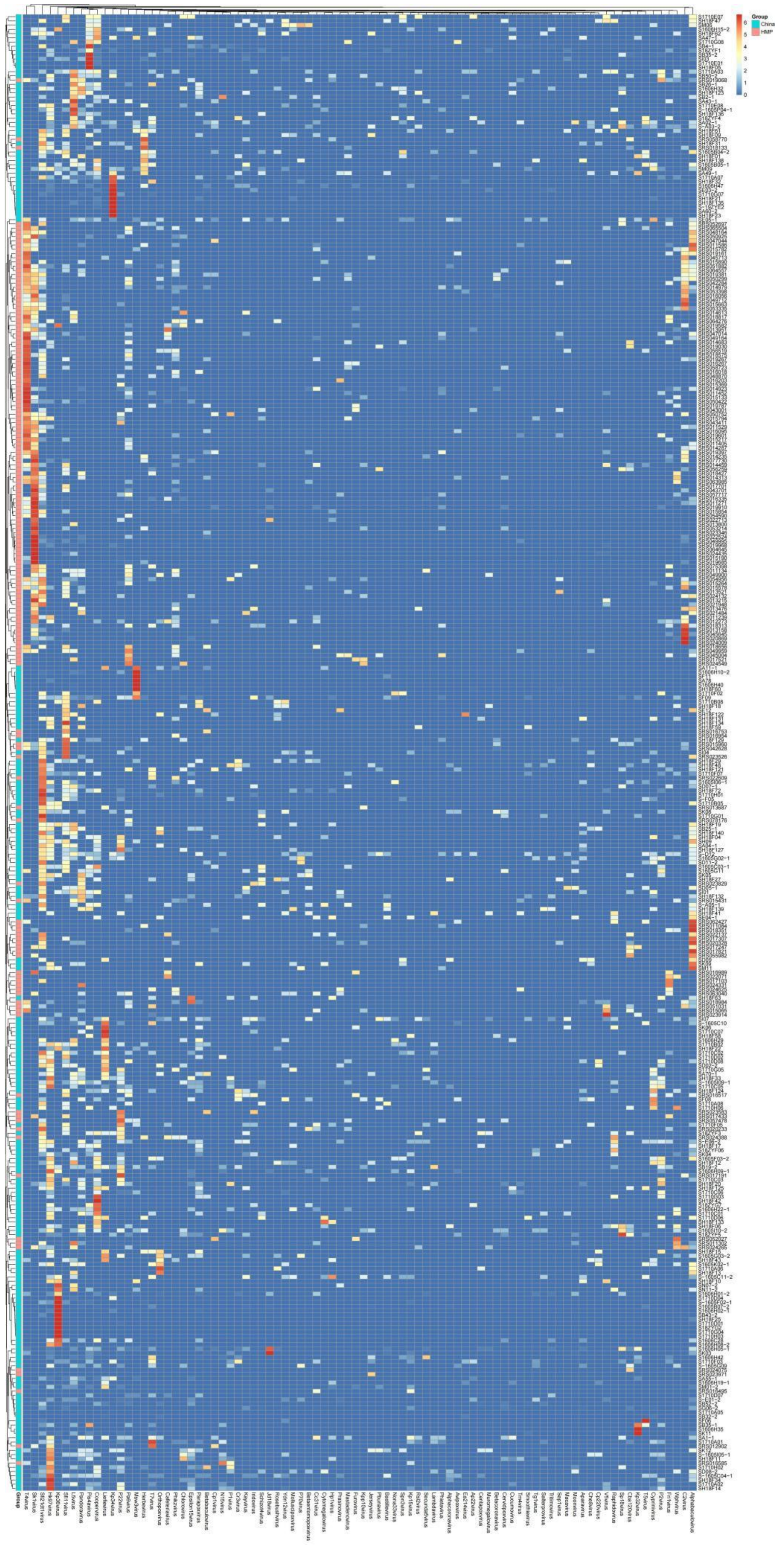

Supplemental Figure 2. The taxa of 149 ARGs on phylum level.

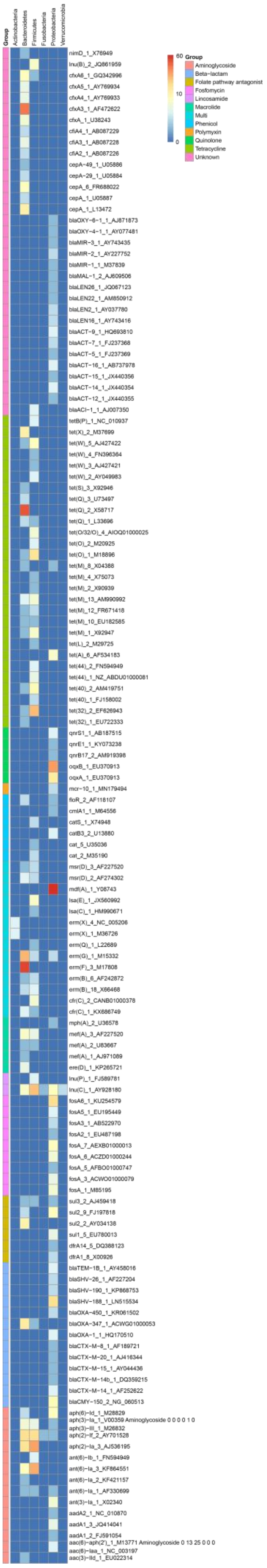

Supplemental Figure 3. KO functional categories identified in CMP dataset.

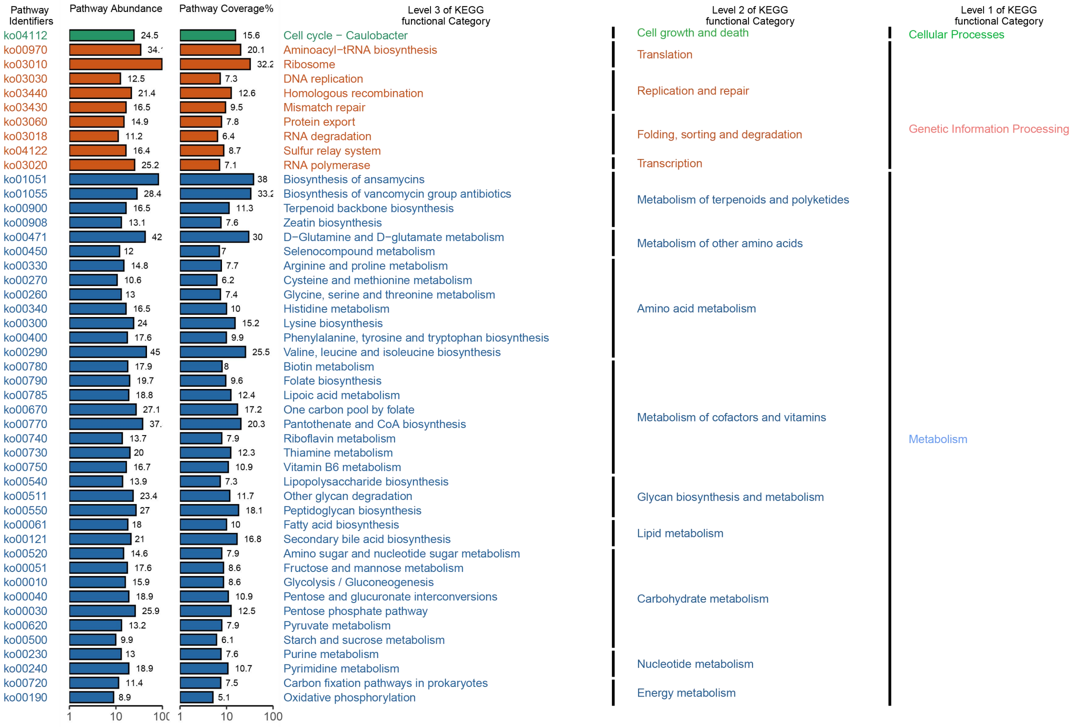

Supplemental Figure 4. (A) The box plots for the richness, Shannon diversity indices, and Pielou evenness index at the genus levels for samples from 4 regions; (B-D) Violin plots presenting the Bray-Curtis dissimilarity between samples from different groups. (E) PCoA diagram of samples from four regions. (F) Violin plots presenting the Genetic similarity (GS) value between samples from different groups.

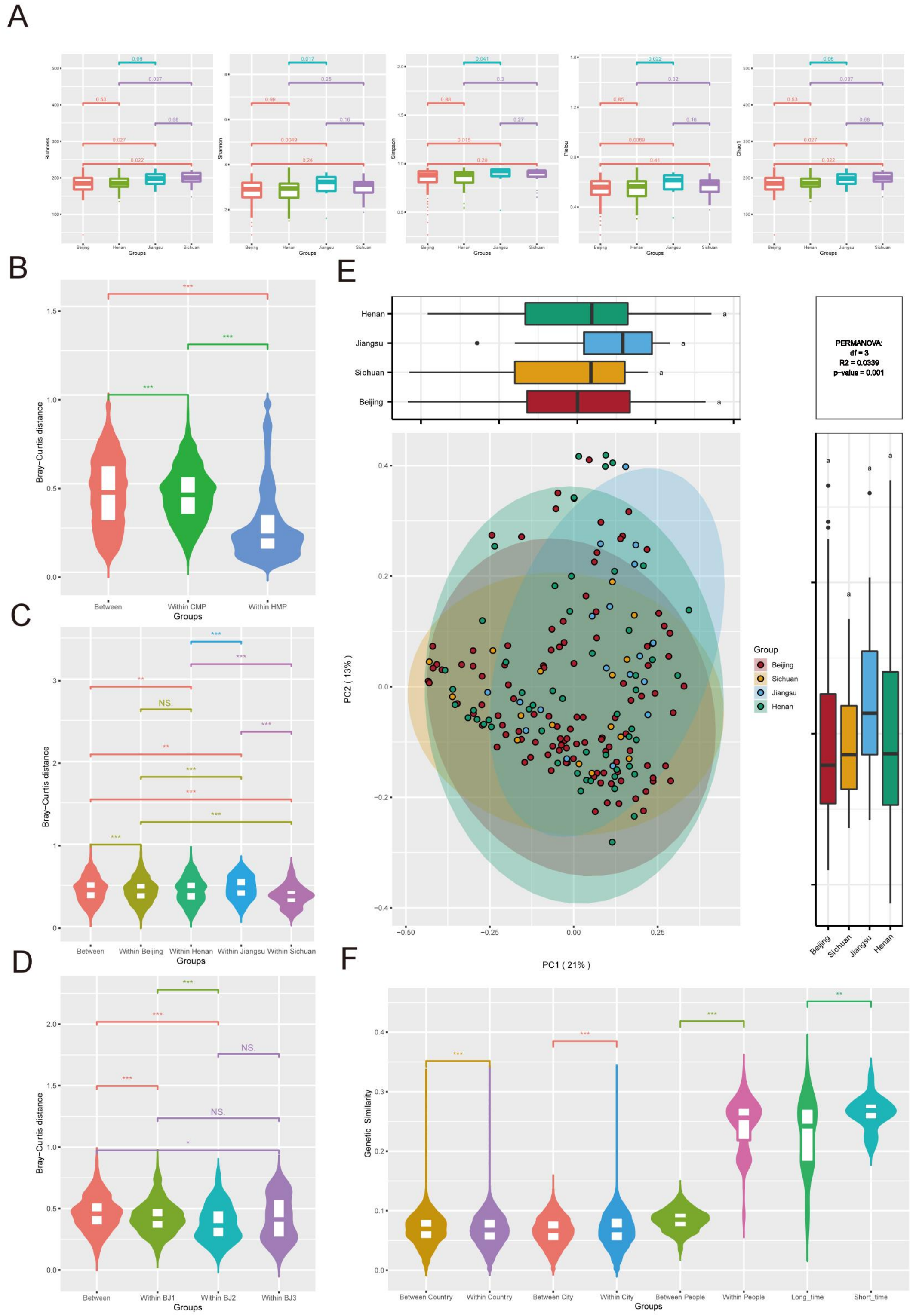

Supplemental Figure 5. The heatmap of ARGs in CMP dataset.

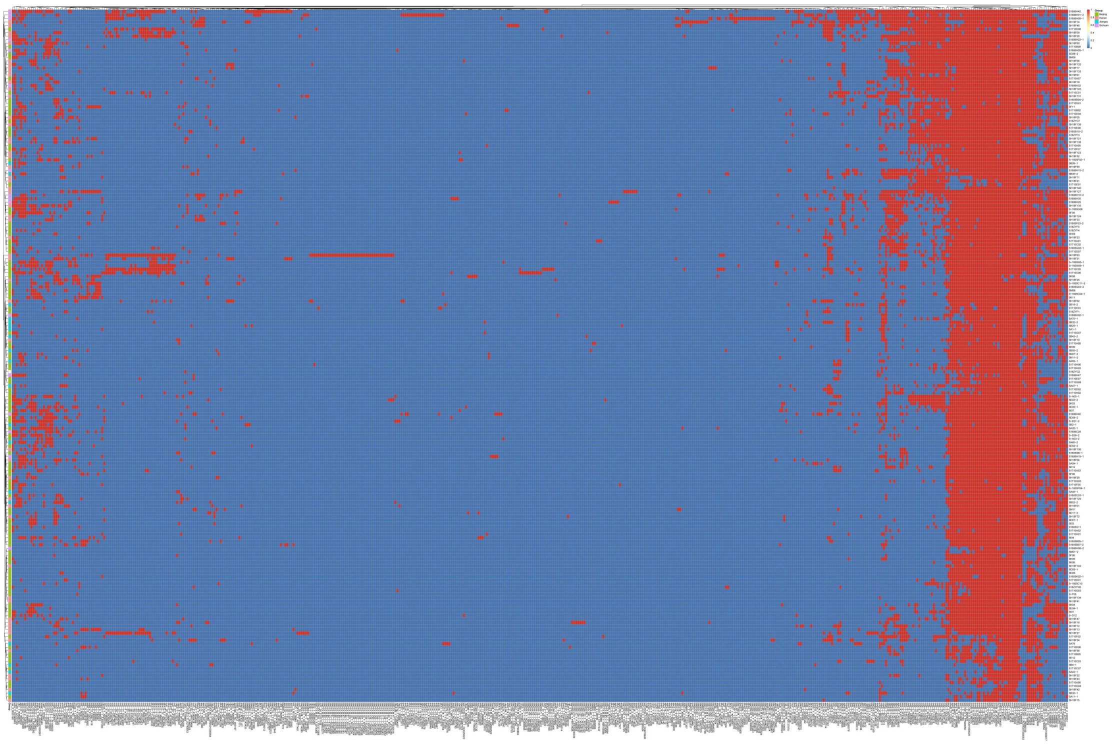

Supplemental Figure 6. The percentage of *Bifidobacterium* genus in Yogurt+ and Yogurt- group.

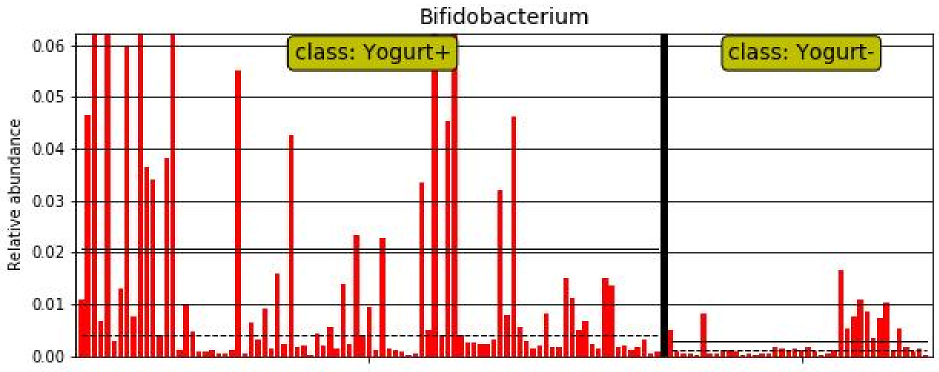

Supplemental Figure 7. The percentage of *Bifidobacterium* genus in Female and Male group.

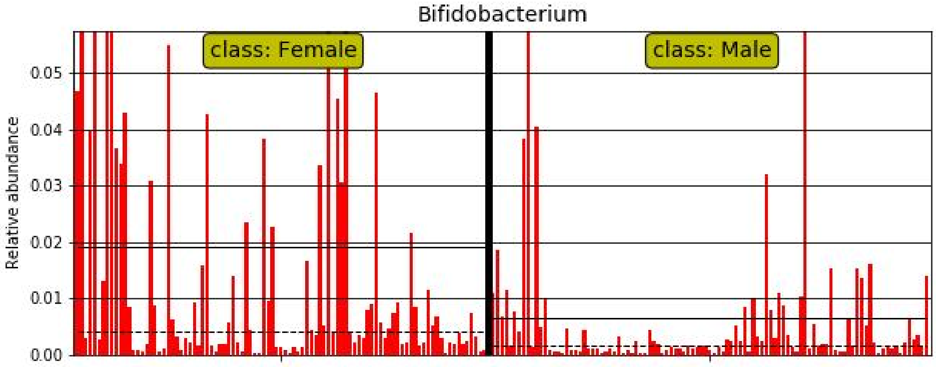

Supplemental Table 1. Detailed Information about 239 samples. Smoke-: Never; Smoke +: More than 2 cigarettes a day; Drink-: Never; Drink+:Every day/Once per two or three days; PE-: Never; PE+: More than 1 hour per day; Yogurt-:Never; Yogurt+:Every day/Three or more times; Fruit-: Never; Fruit+:Every day.

Sample ID	City	Gender	Age	BMI	Smoke	Drink	Physical experience	Yogurt	Fruit
S1606H32	Sichuan	Male	51-70	16-20	Smoke+	Drink-	PE+	Yogurt-	Fruit-
S1606H35	Sichuan	Male	51-70	20-25	Smoke+	Drink-	PE+	Yogurt-	Fruit-
S1606H40	Sichuan	Female	51-70	16-20	Smoke-	Drink-	PE-	Yogurt-	Fruit-
SB25-1	Jiangsu	Male	51-70	20-25	Smoke+	Drink+	PE+	Yogurt-	Fruit-
SB32-2	Jiangsu	Male	18-50	20-25	Smoke+	Drink+	PE+	Yogurt-	Fruit-
S1605I10-2	Beijing	Male	18-50	25-30	Smoke-	Drink-		Yogurt-	
S1606H05-1	Sichuan	Male	18-50	20-25	Smoke-			Yogurt-	
S1606H15-2	Sichuan	Female	18-50	25-30	Smoke-	Drink-	PE-	Yogurt-	
S1606H19-1	Sichuan	Male	18-50	20-25	Smoke+	Drink-	PE-	Yogurt-	
SA43-1	Jiangsu	Male	51-70	20-25	Smoke+		PE-	Yogurt-	
S1710A08	Beijing	Male	18-50	16-20	Smoke-	Drink-		Yogurt-	
S1710D06	Beijing	Male	18-50	20-25	Smoke-	Drink-		Yogurt-	
SH18F129	Henan	Male	18-50	20-25	Smoke-	Drink-	PE+	Yogurt-	
SH18F18	Henan	Male	18-50	16-20	Smoke-	Drink-		Yogurt-	
S1605G02-1	Beijing	Female	18-50	20-25	Smoke-	Drink-	PE+		
S1605I06-1	Beijing	Male	18-50	25-30	Smoke-				
S-A03-2	Beijing	Female	18-50	20-25	Smoke-	Drink-	PE-		
SD02-2	Beijing	Female	18-50	16-20	Smoke-	Drink-			
SD05-1	Beijing	Female	18-50	16-20	Smoke-	Drink-	PE-		
SE04-1	Beijing	Female	18-50	20-25	Smoke-	Drink-			
SG06-2	Beijing	Male	18-50	20-25	Smoke-	Drink-			
SK05	Beijing	Male	18-50	20-25	Smoke-		PE+		
SK06	Beijing	Male	18-50	16-20	Smoke-		PE+		
SK09	Beijing	Male	18-50	20-25	Smoke-				
SK11	Beijing	Male	18-50	20-25	Smoke-				
S1710A02	Beijing	Female	18-50	16-20	Smoke-	Drink-			
S1710A03	Beijing	Female	18-50	20-25	Smoke-				
S1710B05	Beijing	Male	18-50	20-25	Smoke-				
S1710C06	Beijing	Female	18-50	16-20	Smoke-	Drink-			
S1710E07	Beijing	Male	18-50	20-25	Smoke-				
S1710F07	Beijing	Female	18-50	16-20	Smoke-	Drink-			
S1710G08	Beijing	Male	18-50	20-25	Smoke-				

S1710H01	Beijing	Male	18-50	20-25	Smoke-	Drink-			
S1710H03	Beijing	Male	18-50	20-25	Smoke-				
SH18F05	Henan	Male	18-50	25-30	Smoke-	Drink-			
SH18F06	Henan	Male	18-50	16-20					
SH18F123	Henan	Female	18-50	20-25	Smoke-	Drink-			
SH18F136	Henan	Female	18-50	20-25					
SH18F140	Henan	Female	18-50	16-20	Smoke-	Drink-			
SH18F27	Henan	Female	18-50	16-20	Smoke-	Drink-			
SH18F33	Henan	Female	18-50	25-30	Smoke-	Drink-			
SH18F43	Henan	Male	18-50	20-25	Smoke-	Drink-			
SH18F47	Henan	Male	18-50	20-25	Smoke-	Drink-	PE+		
SH18F62	Henan	Female	18-50	20-25	Smoke-	Drink-			
SH18F72	Henan	Female	18-50	20-25	Smoke-	Drink-			
S18ZYF5	Beijing	Female	18-50	20-25	Smoke-	Drink-			
S18ZYH2	Beijing	Female	18-50	20-25	Smoke-	Drink-			
S18ZYH5	Beijing	Female	18-50	20-25	Smoke-	Drink-			
S18ZYI5	Beijing	Female	18-50	20-25	Smoke-	Drink-			
S18ZYK3	Beijing	Female	18-50	20-25	Smoke-	Drink-			
S18ZYK5	Beijing	Female	18-50	20-25	Smoke-	Drink-			
S19ZYB5	Beijing	Female	18-50	20-25	Smoke-	Drink-			
S19ZYC5	Beijing	Female	18-50	20-25	Smoke-	Drink-			
S19ZYE5	Beijing	Female	18-50	20-25	Smoke-	Drink-			
S19ZYF07	Beijing	Female	18-50	20-25	Smoke-	Drink-			
S1605C03-1	Beijing	Male	18-50	20-25	Smoke+		PE-	Yogurt+	
S1605G03-2	Beijing	Male	18-50	20-25	Smoke-			Yogurt+	
S-1605I05-1	Beijing	Male	18-50	25-30	Smoke-			Yogurt+	
SA04-1	Beijing	Female	18-50	16-20	Smoke-	Drink-		Yogurt+	
S-E01-2	Beijing	Female	18-50	20-25	Smoke-	Drink-		Yogurt+	
SE03-2	Beijing	Female	18-50	16-20	Smoke-	Drink-		Yogurt+	
SE05-1	Beijing	Female	18-50	20-25	Smoke-	Drink-		Yogurt+	
SF11	Beijing	Male	18-50	20-25				Yogurt+	
SM06	Beijing	Male	18-50	20-25	Smoke-			Yogurt+	
SM09	Beijing	Female	18-50	16-20	Smoke-			Yogurt+	
S1606H08-2	Sichuan	Male	18-50	20-25	Smoke-	Drink-	PE+	Yogurt+	
S1606H09-1	Sichuan	Male	18-50	16-20	Smoke-	Drink-		Yogurt+	
S1606H10-2	Sichuan	Male	51-70	20-25	Smoke+	Drink+		Yogurt+	
S1710B02	Beijing	Female	18-50	20-25	Smoke-	Drink-	PE+	Yogurt+	

S1710B08	Beijing	Male	18-50	20-25	Smoke-			Yogurt+	
S1710C01	Beijing	Female	18-50	20-25	Smoke-			Yogurt+	
S1710C05	Beijing	Female	18-50	20-25	Smoke-	Drink-		Yogurt+	
S1710C07	Beijing	Female	18-50	16-20	Smoke-	Drink-		Yogurt+	
S1710E08	Beijing	Female	18-50	16-20	Smoke-	Drink-		Yogurt+	
S1710F02	Beijing	Female	18-50	16-20	Smoke-	Drink-		Yogurt+	
S1710F05	Beijing	Female	18-50	20-25	Smoke-	Drink-		Yogurt+	
S1710G07	Beijing	Female	18-50	20-25	Smoke-			Yogurt+	
SH18F11	Henan	Male	18-50	16-20				Yogurt+	
SH18F131	Henan	Female	18-50	20-25	Smoke-	Drink-		Yogurt+	
SH18F133	Henan	Male	18-50	20-25	Smoke-	Drink-		Yogurt+	
SH18F21	Henan	Female	18-50	20-25	Smoke-	Drink-		Yogurt+	
SH18F25	Henan	Male	18-50	20-25			PE+	Yogurt+	
SH18F29	Henan	Female	18-50	16-20	Smoke-	Drink-	PE-	Yogurt+	
SH18F63	Henan	Female	18-50	20-25	Smoke-	Drink-		Yogurt+	
S18ZYF3	Beijing	Female	18-50	20-25	Smoke-	Drink-		Yogurt+	
S18ZYH3	Beijing	Female	18-50	20-25	Smoke-	Drink-		Yogurt+	
S18ZYJ3	Beijing	Female	18-50	20-25	Smoke-	Drink-		Yogurt+	
S19ZYD3	Beijing	Female	18-50	20-25	Smoke-	Drink-		Yogurt+	
SB43-2	Jiangsu	Male	18-50	25-30		Drink-	PE+	Yogurt+	
SH18F48	Henan	Female	18-50	25-30	Smoke-	Drink-		Yogurt+	
SK07	Beijing	Male	18-50	20-25	Smoke-	Drink-	PE+	Yogurt-	Fruit+
S1606C28	Sichuan	Male	51-70	20-25	Smoke-	Drink-		Yogurt-	Fruit+
SB35-1	Jiangsu	Male	51-70	25-30	Smoke+	Drink-	PE+	Yogurt-	Fruit+
SB35-2	Jiangsu	Female	51-70	25-30	Smoke-	Drink-	PE+	Yogurt-	Fruit+
S18ZYF1	Beijing	Female	18-50	20-25	Smoke-	Drink-		Yogurt-	Fruit+
S18ZYG1	Beijing	Female	18-50	20-25	Smoke-	Drink-		Yogurt-	Fruit+
S18ZYH1	Beijing	Female	18-50	20-25	Smoke-	Drink-		Yogurt-	Fruit+
S18ZYI1	Beijing	Female	18-50	20-25	Smoke-	Drink-		Yogurt-	Fruit+
S18ZYK1	Beijing	Female	18-50	20-25	Smoke-	Drink-		Yogurt-	Fruit+
S19ZYB2	Beijing	Female	18-50	20-25	Smoke-	Drink-		Yogurt-	Fruit+
S19ZYD2	Beijing	Female	18-50	20-25	Smoke-	Drink-		Yogurt-	Fruit+
S-19ZYE2	Beijing	Female	18-50	20-25	Smoke-	Drink-		Yogurt-	Fruit+
SM01-2	Beijing	Male	18-50	16-20	Smoke-	Drink-			Fruit+
SH18F04	Henan	Female	18-50	20-25	Smoke-	Drink-	PE-		Fruit+
SH18F132	Henan	Female	18-50	16-20	Smoke-	Drink-			Fruit+
SH18F34	Henan	Female	18-50	20-25	Smoke-	Drink-			Fruit+
S18ZYG7	Beijing	Female	18-50	20-25	Smoke-	Drink-			Fruit+
S18ZYH07	Beijing	Female	18-50	20-25	Smoke-	Drink-			Fruit+
S18ZYI07	Beijing	Female	18-50	20-25	Smoke-	Drink-			Fruit+
S18ZYJ07	Beijing	Female	18-50	20-25	Smoke-	Drink-			Fruit+
S19ZYC2	Beijing	Female	18-50	20-25	Smoke-	Drink-			Fruit+
S19ZYC4	Beijing	Female	18-50	20-25	Smoke-	Drink-			Fruit+

S19ZYD4	Beijing	Female	18-50	20-25	Smoke-	Drink-			Fruit+
S1605B04-2	Beijing	Female	18-50	20-25	Smoke-	Drink-		Yogurt+	Fruit+
S1605B05-1	Beijing	Female	18-50	20-25	Smoke-	Drink-		Yogurt+	Fruit+
S-1605C04-1	Beijing	Male	18-50	20-25	Smoke-			Yogurt+	Fruit+
S-1605C10	Beijing	Female	18-50	20-25	Smoke-	Drink+		Yogurt+	Fruit+
S1605F03-2	Beijing	Male	18-50	20-25	Smoke-	Drink-		Yogurt+	Fruit+
S1605G11	Beijing	Male	18-50	20-25	Smoke-			Yogurt+	Fruit+
SD07-1	Beijing	Female	18-50	20-25	Smoke-	Drink-		Yogurt+	Fruit+
SD09	Beijing	Female	18-50	20-25	Smoke-	Drink-		Yogurt+	Fruit+
S-E06-2	Beijing	Female	18-50	16-20	Smoke-	Drink-	PE-	Yogurt+	Fruit+
SF06	Beijing	Male	18-50	20-25	Smoke-	Drink-		Yogurt+	Fruit+
SH09	Beijing	Female	18-50	20-25	Smoke-	Drink-		Yogurt+	Fruit+
SN07-2	Beijing	Female	18-50	20-25	Smoke-			Yogurt+	Fruit+
SN11-2	Beijing	Female	18-50	20-25	Smoke-	Drink-		Yogurt+	Fruit+
SA47-1	Jiangsu	Female	18-50	20-25	Smoke-	Drink-		Yogurt+	Fruit+
SA70-1	Jiangsu	Female	18-50	16-20	Smoke-	Drink-		Yogurt+	Fruit+
S1710A01	Beijing	Female	18-50	20-25	Smoke-	Drink-		Yogurt+	Fruit+
S1710A06	Beijing	Female	18-50	20-25	Smoke-	Drink-	PE+	Yogurt+	Fruit+
S1710A07	Beijing	Female	18-50	20-25	Smoke-	Drink-		Yogurt+	Fruit+
S1710D04	Beijing	Female	18-50	20-25	Smoke-	Drink-		Yogurt+	Fruit+
S1710G05	Beijing	Male	18-50	20-25	Smoke+			Yogurt+	Fruit+
SH18F121	Henan	Female	18-50	20-25	Smoke-	Drink-		Yogurt+	Fruit+
SH18F32	Henan	Female	18-50	20-25	Smoke-	Drink-		Yogurt+	Fruit+
S18ZYF06	Beijing	Female	18-50	20-25	Smoke-	Drink-		Yogurt+	Fruit+
S18ZYF4	Beijing	Female	18-50	20-25	Smoke-	Drink-		Yogurt+	Fruit+
S18ZYG2	Beijing	Female	18-50	20-25	Smoke-	Drink-		Yogurt+	Fruit+
S18ZYG6	Beijing	Female	18-50	20-25	Smoke-	Drink-		Yogurt+	Fruit+
S18ZYH4	Beijing	Female	18-50	20-25	Smoke-	Drink-		Yogurt+	Fruit+
S18ZYH6	Beijing	Female	18-50	20-25	Smoke-	Drink-		Yogurt+	Fruit+
S18ZYI6	Beijing	Female	18-50	20-25	Smoke-	Drink-		Yogurt+	Fruit+
S18ZYJ2	Beijing	Female	18-50	20-25	Smoke-	Drink-		Yogurt+	Fruit+
S18ZYJ6	Beijing	Female	18-50	20-25	Smoke-	Drink-		Yogurt+	Fruit+
S18ZYK2	Beijing	Female	18-50	20-25	Smoke-	Drink-		Yogurt+	Fruit+
S18ZYK6	Beijing	Female	18-50	20-25	Smoke-	Drink-		Yogurt+	Fruit+
S19ZYA1	Beijing	Female	51-70	20-25	Smoke-	Drink-		Yogurt+	Fruit+
S19ZYB1	Beijing	Female	51-70	20-25	Smoke-	Drink-		Yogurt+	Fruit+
S19ZYC1	Beijing	Female	51-70	20-25	Smoke-	Drink-		Yogurt+	Fruit+
S19ZYD1	Beijing	Female	51-70	20-25	Smoke-	Drink-		Yogurt+	Fruit+
S19ZYE1	Beijing	Female	51-70	20-25	Smoke-	Drink-		Yogurt+	Fruit+

SZY19A4	Beijing	Female	18-50	20-25	Smoke-	Drink-		Yogurt+	Fruit+
SZY19B4	Beijing	Female	18-50	20-25	Smoke-	Drink-		Yogurt+	Fruit+
S-1605C11-2	Beijing	Female	51-70	20-25	Smoke-		PE-	Yogurt+	Fruit+
SE12	Beijing	Female	18-50	16-20	Smoke-	Drink-		Yogurt+	Fruit+
SA49-1	Jiangsu	Female	18-50	16-20	Smoke-	Drink-		Yogurt+	Fruit+
SH18F09	Henan	Male	18-50	16-20	Smoke-	Drink-	PE+	Yogurt+	Fruit+
S18ZYJ1	Beijing	Female	18-50	20-25	Smoke-	Drink-		Yogurt+	Fruit+
SI03	Beijing	Male	18-50	20-25	Smoke-	Drink-	PE+		Fruit+
SF09	Beijing	Male	18-50	20-25	Smoke-			Yogurt-	
SK08	Beijing	Male	18-50	20-25	Smoke-		PE+	Yogurt-	
S1606H01-2	Sichuan	Male	18-50	20-25	Smoke+	Drink-		Yogurt-	
S1606H02-1	Sichuan	Male	18-50	25-30	Smoke+			Yogurt-	
S1606H29	Sichuan	Female	18-50	25-30	Smoke-	Drink-	PE-	Yogurt-	
S1606H47	Sichuan	Male	18-50	25-30	Smoke+			Yogurt-	
SA60-2	Jiangsu	Female	51-70	16-20	Smoke-	Drink-	PE-	Yogurt-	
SB19-2	Jiangsu	Female	51-70	20-25			PE+	Yogurt-	
SB2-1	Jiangsu	Male	51-70	20-25	Smoke+	Drink+	PE+	Yogurt-	
SB26-1	Jiangsu	Male	51-70	20-25	Smoke+	Drink+	PE+	Yogurt-	
SB50-2	Jiangsu	Female	18-50	20-25	Smoke-	Drink-	PE+	Yogurt-	
SB52-2	Jiangsu	Female	51-70	25-30	Smoke-	Drink-	PE+	Yogurt-	
S1710F03	Beijing	Female	18-50	20-25	Smoke-	Drink-	PE+	Yogurt-	
SH18F12	Henan	Male	18-50	20-25				Yogurt-	
SH18F127	Henan	Male	18-50	16-20				Yogurt-	
SH18F13	Henan	Male	18-50	16-20	Smoke-	Drink-	PE+	Yogurt-	
SH18F134	Henan	Female	18-50	20-25	Smoke-	Drink-		Yogurt-	
SH18F14	Henan	Male	18-50	20-25				Yogurt-	
SH18F17	Henan	Male	18-50	20-25	Smoke-	Drink-		Yogurt-	
SH18F19	Henan	Male	18-50	16-20	Smoke-	Drink-		Yogurt-	
SH18F41	Henan	Male	18-50	16-20	Smoke-	Drink-		Yogurt-	
S-1605F02-1	Beijing	Male	18-50	20-25	Smoke-	Drink-			
S-1605I09-1	Beijing	Male	18-50	20-25		Drink-			
S-1605P04-1	Beijing	Male	18-50	25-30	Smoke-				
SA02-1	Beijing	Female	18-50	16-20	Smoke-	Drink-	PE-		
SA11-1	Beijing	Male	18-50	25-30	Smoke-				
SI01	Beijing	Female	18-50	20-25	Smoke-	Drink-			
SI04	Beijing	Male	18-50	25-30					
SI07	Beijing	Male	18-50	16-20	Smoke-				

SK03	Beijing	Male	18-50	20-25	Smoke-	Drink-			
SK12	Beijing	Male	18-50	20-25	Smoke-	Drink-			
S1606H22-1	Sichuan	Male	18-50	20-25	Smoke-	Drink-	PE-		
SB4-1	Jiangsu	Male	51-70	20-25	Smoke+	Drink-	PE+		
S1710A05	Beijing	Female	18-50	20-25	Smoke-	Drink-			
S1710C02	Beijing	Female	18-50	20-25	Smoke-				
S1710C03	Beijing	Female	18-50	16-20	Smoke-				
S1710D01	Beijing	Male	18-50	20-25	Smoke-	Drink-			
S1710D07	Beijing	Male	18-50	20-25	Smoke-	Drink-			
S1710D08	Beijing	Male	18-50	20-25	Smoke-	Drink-			
S1710G04	Beijing	Male	18-50	20-25	Smoke-				
SH18F01	Henan	Female	18-50	16-20	Smoke-	Drink-			
SH18F10	Henan	Male	18-50	20-25	Smoke-	Drink-			
SH18F122	Henan	Male	18-50	20-25			PE+		
SH18F135	Henan	Female	18-50	20-25	Smoke-	Drink-			
SH18F138	Henan	Male	18-50	20-25	Smoke-	Drink-			
SH18F15	Henan	Male	18-50	20-25					
SH18F20	Henan	Male	18-50	20-25	Smoke-	Drink-	PE+		
SH18F42	Henan	Male	18-50	20-25	Smoke-	Drink-	PE+		
S1605B07-2	Beijing	Female	18-50	20-25	Smoke-			Yogurt+	
S1605K02-1	Beijing	Male	18-50	20-25				Yogurt+	
SD08-2	Beijing	Female	18-50	16-20	Smoke-	Drink-		Yogurt+	
SD11-2	Beijing	Female	18-50	16-20	Smoke-	Drink-		Yogurt+	
S-D12	Beijing	Female	18-50	16-20	Smoke-	Drink-		Yogurt+	
S-F05	Beijing	Male	18-50	16-20	Smoke-	Drink-	PE+	Yogurt+	
SF08	Beijing	Male	18-50	20-25	Smoke-			Yogurt+	
SK04	Beijing	Male	18-50	25-30	Smoke+			Yogurt+	
S1710D02	Beijing	Female	18-50	20-25	Smoke-	Drink-		Yogurt+	
S1710E01	Beijing	Male	18-50	25-30	Smoke-		PE-	Yogurt+	
S1710G01	Beijing	Male	18-50	20-25	Smoke-	Drink-		Yogurt+	
S1710G03	Beijing	Male	18-50	20-25	Smoke-			Yogurt+	
S1710H02	Beijing	Male	18-50	20-25				Yogurt+	
S1710H06	Beijing	Female	18-50	16-20	Smoke-	Drink-		Yogurt+	
SH18F124	Henan	Female	18-50	16-20				Yogurt+	
SH18F125	Henan	Female	18-50	20-25	Smoke-	Drink-		Yogurt+	
SH18F139	Henan	Female	18-50	20-25	Smoke-	Drink-	PE+	Yogurt+	
SH18F22	Henan	Female	18-50	20-25	Smoke-	Drink-	PE+	Yogurt+	
SH18F23	Henan	Male	18-50	20-25	Smoke-	Drink-		Yogurt+	
SH18F31	Henan	Female	18-50	20-25	Smoke-	Drink-		Yogurt+	
SH18F58	Henan	Male	18-50	20-25	Smoke-	Drink-	PE+	Yogurt+	

SH18F59	Henan	Male	18-50	20-25	Smoke-	Drink-		Yogurt+	
SH18F60	Henan	Male	18-50	16-20	Smoke-	Drink-		Yogurt+	
SH18F61	Henan	Female	18-50	20-25	Smoke-	Drink-		Yogurt+	
S-A05-1	Beijing	Female	18-50	20-25	Smoke-	Drink-		Yogurt+	
S1606H42	Sichuan	Male	51-70	20-25	Smoke-	Drink-		Yogurt+	
SA1-1	Jiangsu	Male	51-70	20-25	Smoke+	Drink+	PE-	Yogurt+	
SA55-1	Jiangsu	Male	51-70		Smoke+	Drink+	PE+	Yogurt+	
SA78	Jiangsu	Female	51-70	20-25	Smoke+	Drink-	PE+	Yogurt+	
SM11	Beijing	Male	18-50	20-25	Smoke-	Drink-	PE-		
S18ZYJ5	Beijing	Female	18-50	20-25	Smoke-	Drink-			
S-1605G09	Beijing	Female	18-50	20-25					

Supplemental Table 2. The percentage of these DRMs in HMP and CMP projects.

DRM	Group	HMP pos %	CMP pos %
Mycobacterium leprae	Group1	0.0%	0.0%
Bartonella bacilliformis	Group1	0.0%	0.0%
Campylobacter fetus	Group1	0.0%	0.0%
Borrelia recurrentis	Group1	0.0%	0.0%
Vibrio fluvialis	Group1	0.0%	0.0%
Streptobacillus moniliformis	Group1	0.0%	0.0%
Serratia marcescens	Group1	0.0%	0.0%
Borrelia duttonii	Group1	0.0%	0.0%
Burkholderia mallei	Group1	0.0%	0.0%
Listeria monocytogenes	Group1	0.0%	0.0%
Treponema pallidum	Group1	0.0%	0.0%
Mycobacterium kansasii	Group1	0.0%	0.0%
Streptococcus iniae	Group1	0.0%	0.0%
Mycobacterium tuberculosis	Group1	0.0%	0.0%
Corynebacterium diphtheriae	Group1	0.0%	0.0%
Mycobacterium simiae	Group1	0.0%	0.0%
Streptococcus merionis	Group1	0.0%	0.0%
Eikenella corrodens	Group1	0.0%	0.0%
Clostridium tetani	Group1	0.0%	0.0%
Nocardia nova	Group1	0.0%	0.0%
Yersinia enterocolitica	Group1	0.0%	0.0%
Bartonella vinsonii	Group1	0.0%	0.0%
Morganella morganii	Group1	0.0%	0.0%
Streptococcus mutans	Group1	0.0%	0.0%
Mycobacterium avium	Group1	0.0%	0.0%
Streptococcus ferus	Group1	0.0%	0.0%
Rickettsia monacensis	Group1	0.0%	0.0%
Brachyspira pilosicoli	Group1	0.0%	0.0%
Rickettsia helvetica	Group1	0.0%	0.0%

Anaplasma phagocytophilum	Group1	0.0%	0.0%
Clostridium novyi	Group1	0.0%	0.0%
Providencia alcalifaciens	Group1	0.0%	0.0%
Brucella canis	Group1	0.0%	0.0%
Acinetobacter baumannii	Group1	0.0%	0.0%
Vibrio mimicus	Group1	0.0%	0.0%
Fusobacterium necrophorum	Group1	0.0%	0.0%
Vibrio vulnificus	Group1	0.0%	0.0%
Campylobacter sputorum	Group1	0.0%	0.0%
Yersinia pseudotuberculosis	Group1	0.0%	0.0%
Streptococcus australis	Group1	0.0%	0.0%
Streptococcus uberis	Group1	0.0%	0.0%
Arcanobacterium haemolyticum	Group1	0.0%	0.0%
Streptococcus parauberis	Group1	0.0%	0.0%
Legionella pneumophila	Group1	0.0%	0.0%
Mycoplasma pneumoniae	Group1	0.0%	0.0%
Listeria ivanovii	Group1	0.0%	0.0%
Rickettsia sibirica	Group1	0.0%	0.0%
Rickettsia massiliae	Group1	0.0%	0.0%
Streptococcus sobrinus	Group1	0.0%	0.0%
Providencia rettgeri	Group1	0.0%	0.0%
Corynebacterium minutissimum	Group1	0.0%	0.0%
Streptococcus equi	Group1	0.0%	0.0%
Nocardia brasiliensis	Group1	0.0%	0.0%
Bordetella bronchiseptica	Group1	0.0%	0.0%
Rickettsia akari	Group1	0.0%	0.0%
Corynebacterium pseudotuberculosis	Group1	0.0%	0.0%
Brachyspira hyodysenteriae	Group1	0.0%	0.0%
Neorickettsia sennetsu	Group1	0.0%	0.0%
Streptococcus acidominimus	Group1	0.0%	0.0%
Streptococcus gallolyticus	Group1	0.0%	0.0%
Rickettsia canadensis	Group1	0.0%	0.0%
Vibrio cholerae	Group1	0.0%	0.0%
Chlamydia trachomatis	Group1	0.0%	0.0%
Dermatophilus congolensis	Group1	0.0%	0.0%
Pasteurella multocida	Group1	0.0%	0.0%
Nocardia farcinica	Group1	0.0%	0.0%
Streptococcus intermedius	Group1	0.0%	0.0%
Mycobacterium ulcerans	Group1	0.0%	0.0%
Streptococcus gordonii	Group1	0.0%	0.0%
Francisella tularensis	Group1	0.0%	0.0%
Shigella flexneri	Group1	0.0%	0.0%
Ureaplasma urealyticum	Group1	0.0%	0.0%

Bordetella pertussis	Group1	0.0%	0.0%
Rickettsia conorii	Group1	0.0%	0.0%
Yersinia pestis	Group1	0.0%	0.0%
Brachyspira murdochii	Group1	0.0%	0.0%
Brachyspira intermedia	Group1	0.0%	0.0%
Brucella ovis	Group1	0.0%	0.0%
Rickettsia japonica	Group1	0.0%	0.0%
Streptococcus anginosus	Group1	0.0%	0.0%
Neisseria gonorrhoeae	Group1	0.0%	0.0%
Kingella kingae	Group1	0.0%	0.0%
Bartonella quintana	Group1	0.0%	0.0%
Streptococcus dysgalactiae	Group1	0.0%	0.0%
Streptococcus pyogenes	Group1	0.0%	0.0%
Bordetella parapertussis	Group1	0.0%	0.0%
Streptococcus equinus	Group1	0.0%	0.0%
Trueperella pyogenes	Group1	0.0%	0.0%
Corynebacterium ulcerans	Group1	0.0%	0.0%
Proteus vulgaris	Group1	0.0%	0.0%
Bartonella henselae	Group1	0.0%	0.0%
Elizabethkingia meningoseptica	Group1	0.0%	0.0%
Pseudomonas aeruginosa	Group1	0.0%	0.0%
Streptococcus porcinus	Group1	0.0%	0.0%
Gardnerella vaginalis	Group1	0.0%	0.0%
Erysipelothrix rhusiopathiae	Group1	0.0%	0.0%
Helicobacter pylori	Group1	0.0%	0.0%
Proteus mirabilis	Group1	0.0%	0.0%
Ehrlichia chaffeensis	Group1	0.0%	0.0%
Leptospira interrogans	Group1	0.0%	0.0%
Coxiella burnetii	Group1	0.0%	0.0%
Acinetobacter lwoffii	Group1	0.0%	0.0%
Streptococcus pluranimalium	Group1	0.0%	0.0%
Aggregatibacter actinomycetemcomitans	Group1	0.0%	0.0%
Streptococcus infantarius	Group1	0.0%	0.0%
Streptococcus constellatus	Group1	0.0%	0.0%
Serratia liquefaciens	Group1	0.0%	0.0%
Bacillus anthracis	Group1	0.0%	0.0%
Staphylococcus epidermidis	Group1	0.0%	0.0%
Streptococcus cristatus	Group2	0.0%	0.5%
Streptococcus pseudopneumoniae	Group2	0.0%	0.5%
Edwardsiella tarda	Group2	0.0%	0.5%
Cronobacter sakazakii	Group2	0.0%	0.5%
Streptococcus lutetiensis	Group2	0.0%	0.5%
Enterobacter ludwigii	Group2	0.0%	0.5%

Aeromonas hydrophila	Group2	0.0%	0.5%
Bacillus cereus	Group2	0.0%	0.5%
Plesiomonas shigelloides	Group2	0.0%	0.5%
Neisseria meningitidis	Group2	0.0%	0.5%
Campylobacter coli	Group2	0.0%	0.5%
Streptococcus sanguinis	Group2	0.0%	0.5%
Streptococcus oralis	Group2	0.0%	1.0%
Staphylococcus aureus	Group2	0.0%	1.0%
Clostridium botulinum	Group2	0.0%	1.0%
Haemophilus influenzae	Group2	0.0%	1.5%
Clostridium perfringens	Group2	0.0%	1.5%
Streptococcus pneumoniae	Group2	0.0%	3.0%
Enterobacter kobei	Group2	0.0%	3.5%
Klebsiella oxytoca	Group2	0.0%	4.0%
Enterobacter hormaechei	Group2	0.0%	16.2%
Enterococcus faecalis	Group2	0.7%	0.0%
Shigella dysenteriae	Group2	0.7%	1.0%
Enterobacter asburiae	Group2	0.7%	2.5%
Streptococcus parasanguinis	Group2	0.7%	5.1%
Streptococcus mitis	Group2	0.7%	6.1%
Citrobacter freundii	Group2	0.7%	16.2%
Streptococcus suis	Group2	1.3%	2.0%
Salmonella enterica	Group2	1.3%	22.7%
Enterobacter cloacae	Group2	1.3%	22.7%
Campylobacter jejuni	Group2	1.3%	23.2%
Klebsiella pneumoniae	Group2	1.3%	46.0%
Prevotella dentalis	Group2	2.0%	49.0%
Prevotella denticola	Group2	2.6%	52.0%
Streptococcus agalactiae	Group2	3.3%	5.1%
Prevotella enoeca	Group2	3.3%	46.0%
Prevotella jejuni	Group2	3.9%	44.4%
Enterococcus faecium	Group2	4.6%	5.1%
Prevotella scopos	Group2	5.9%	45.5%
Prevotella ruminicola	Group2	7.2%	52.0%
Prevotella fusca	Group2	7.9%	48.0%
Prevotella melaninogenica	Group2	11.2%	52.5%
Prevotella intermedia	Group2	15.1%	63.6%
Escherichia coli	Group2	31.6%	90.4%
Clostridioides difficile	Group2	62.5%	96.5%
Bacteroides fragilis	Group2	99.3%	100.0%

Supplemental Material 1. Personal Information Questionnaire

Personal Information Questionnaire

- ID:
- **Basic information**

Name		Gender			
Age		Height(cm)		Weight (kg)	
Blood pressure					
Blood sugar after diet					

- **History of drug use or disease in the past month**

	Other
Have you taken antibiotics in the past month (such as cephalosporins, penicillin, etc.)? Yes☐ No☐	
Have you used infusion therapy in the past month? Yes☐ No☐	
Have you had pain, hemorrhoids, or blood in the stool in the past month? Yes☐ No☐	
Have you had a rectal examination or treatment in the past month? Yes☐ No☐	
Have you had a case of diarrhea (watery stool or egg soup-like) in the past month? Yes☐ No☐	
Has there been a period of more than 3 days in the past month when you did not defecate? Yes☐ No☐	
Have you taken any drugs to help defecate in the past month? Yes☐ No☐	
Have you taken any drugs to treat diarrhea in the past month? Yes☐ No☐	
Have you had surgery in the past month? Yes☐ No☐	
Have you taken any other drugs in the past month? Yes☐ No☐	

- **Diet and lifestyle habit survey**

How often have you eaten lactic acid products (such as yogurt) in the past month?	☐ Every day	☐ Three or more times	☐ One or two times	☐Never
How often have you eaten	☐ Every day	☐Once per two	☐Occasionally	☐Never

fruit in the past month?			or three days		
How often have you smoked?	☐More than 2 cigarettes a day		☐Once per two or three days	☐Occasionally	☐Never
How often have you drunk alcohol?	☐ Every day		☐Once per two or three days	☐Occasionally	☐Never
How often have you exercised in the past month	☐ More than 1 hour per day		☐one or two times per week	☐Occasionally	☐Never
How many hours did you sleep in the past month	☐more than 8 hours per one day		☐6-8 hours per day	☐4-6 hours per day	☐<4 hours per day
What do you mainly eat?	☐light	☐salty	☐sweet	☐hot and spicy	☐fried food

- **Working and living environment**

- ☐Noise
- ☐Electromagnetic radiation
- ☐Dust pollution
- ☐Chemical pollution
- ☐Air pollution
- ☐Pollution of building decoration
- ☐Cooking fume pollution
- ☐Biochemical Reagent
- ☐Other pollution
- ☐None of the above

- **Medical History**

☐Hypertension	☐Diabetes	☐Cirrhosis	☐History of obesity	☐Helicobacter pylori infection
☐Coronary heart disease	☐Hyperthyroidism	☐Pancreatic disease	☐Peripheral vascular disease	☐Chronic cholecystitis
☐Rheumatic heart disease	☐Anemia	☐Acute and chronic nephritis	☐Heart failure	☐Chronic breast disease
☐Congenital heart disease	☐Epilepsy	☐Connective tissue disease	☐Chronic obstructive pulmonary disease	☐Blood lipid abnormality
☐Cardiomyopathy	☐Mental Disease	☐Sexually transmitted	☐Osteoporosis	☐Elevated uric acid

		disease		
☐Bronchiectasis	☐Neurosis	☐Cancer	☐Gout	☐History of severe allergies
☐Bronchial asthma	☐History of drug abuse	☐History of surgery	☐Rheumatoid arthritis	☐Hereditary disease
☐Emphysema	☐Acute and chronic hepatitis	☐History of severe trauma	☐Cerebral apoplexy	☐Other disease
☐Peptic ulcer	☐Tuberculosis	☐HPV	☐Asthma	

- **Medical history of immediate family**

☐Hypertension	☐Diabetes	☐Cirrhosis	☐History of obesity	☐Helicobacter pylori infection
☐Coronary heart disease	☐Hyperthyroidism	☐Pancreatic disease	☐Peripheral vascular disease	☐Chronic cholecystitis
☐Rheumatic heart disease	☐Anemia	☐Acute and chronic nephritis	☐Heart failure	☐Chronic breast disease
☐Congenital heart disease	☐Epilepsy	☐Connective tissue disease	☐Chronic obstructive pulmonary disease	☐Blood lipid abnormality
☐Cardiomyopathy	☐Mental Disease	☐Sexually transmitted disease	☐Osteoporosis	☐Elevated uric acid
☐Bronchiectasis	☐Neurosis	☐Cancer	☐Gout	☐History of severe allergies
☐Bronchial asthma	☐History of drug abuse	☐History of surgery	☐Rheumatoid arthritis	☐Hereditary disease
☐Emphysema	☐Acute and chronic hepatitis	☐History of severe trauma	☐Cerebral apoplexy	☐Other disease
☐Peptic ulcer	☐Tuberculosis	☐HPV	☐Asthma	
